# Efficacy and safety of ARX788 for individuals with HER2-positive breast cancer and brain metastases (ACE-Breast-06): a single-arm, phase 2 trial in China

**DOI:** 10.1016/j.eclinm.2025.103614

**Published:** 2025-10-30

**Authors:** Ting Li, Mingxi Lin, Biyun Wang, Mingchuan Zhao, Jun Cao, Leiping Wang, Zhonghua Tao, Juan Jin, Haitao Miao, Chengcheng Gong, Yannan Zhao, Wenxia Peng, Xichun Hu, Jian Zhang

**Affiliations:** a Department of Medical Oncology, Fudan University Shanghai Cancer Center, Shanghai, 200032, China; b Department of Oncology, Shanghai Medical College, Fudan University, Shanghai, 200032, China; c Phase I Clinical Trial Center, Fudan University Shanghai Cancer Center, Shanghai, 200032, China

**Keywords:** HER2-positive, Breast cancer, Brain metastases, ARX788

## Abstract

**Background:**

ARX788 is a next-generation antibody–drug conjugate (ADC) in which an anti-HER2 monoclonal antibody is linked to a monomethyl auristatin F (MMAF) payload. This study aimed to investigate the intracranial efficiency of ARX788 in epidermal growth factor receptor 2 (HER2)-positive breast cancer with active brain metastases.

**Methods:**

This prospective, single-arm, phase 2 trial was conducted at a single centre in Shanghai, China. Eligible participants were patients (aged 18–75 years) with HER2-positive breast cancer who had received trastuzumab, taxane, and tyrosine kinase inhibitor (TKI) treatment, and also had at least one measurable active brain metastatic lesion (≥1 cm). Participants received ARX788 at 1.5 mg/kg via intravenous infusion every 3 weeks until disease progression or unacceptable toxicity. The primary endpoint was clinical benefit rate (CBR) in the central nervous system (CNS), defined as the proportion of patients with complete response (CR)/partial response (PR)/stable disease (SD) at ≥24 weeks as per RANO-BM. Secondary endpoints included CNS overall response rate, CNS progression-free survival (PFS), PFS, overall survival, sites of next progression, and safety. Tumour response was assessed every 6 weeks. Both efficacy and safety analyses included patients who had received at least one dose of ARX788. This study is registered with ClinicalTrials.gov, NCT05018702.

**Findings:**

Between August 2021 and April 2025, 32 patients were included and treated with ARX788. The median follow-up duration was 15.0 months (range, 1.8–42.5), and two patients were still receiving ARX788 treatment at final data cutoff (April 2025). As for the intracranial efficacy, the CNS CBR was 34.4% (95% CI: 18.6–53.2; 11 of 32 patients) and the proportion of patients with confirmed CNS overall response rate was 25.0% (11.5–43.4). Median CNS PFS was 5.6 (95% CI: 3.0–7.4) months and median PFS was 5.5 (95% CI: 2.8–7.0) months. Data on overall survival were immature at the time of data analysis. As for the sites of next progression, 8.3% of participants had extracranial progression alone, 70.8% had intracranial progression alone, and 20.8% had both intracranial and extracranial progression. Grade ≥3 treatment-related adverse events (TRAEs) occurred in four (12.5%) of 32 patients. The most common grade ≥3 TRAEs were blurred vision (9.4%), interstitial lung disease/pneumonitis (6.3%), keratopathy (6.3%), dry eyes (3.1%), and thrombocytopaenia (3.1%). The rate of grade ≥3 gastrointestinal toxicity was less than 1%. A grade 5 ILD/pneumonitis event occurred in one (3.1%) patient.

**Interpretation:**

This phase 2 trial is, to our knowledge, the first study of ARX788 in patients with brain metastases. Whilst acknowledging the limitations of this design, our findings show support for ARX788 as a potential treatment alternative for patients with HER2-positive active brain metastases. Future prospective studies incorporating patient-reported outcomes are needed to better capture the clinical benefit of ARX788.

**Funding:**

National Natural Science Foundation of China; National Key Research and Development Program of China; Shanghai Science and Technology Innovation Action Plan; and Beijing Science and Technology Innovation Medical Development Foundation Key Project.

**Research in context**

**Evidence before this study**

ARX788 is a next-generation antibody–drug conjugates (ADC) in which an anti-HER2 monoclonal antibody is site-specifically linked via the unnatural amino acid para-acetylphenylalanine (pAF) to Amberstatin269 (AS269), a proprietary version of monomethyl auristatin F (MMAF) payload. We conducted a systematic search of PubMed for clinical trials investigating ARX788 in the treatment of breast cancer plus brain metastases (BrMs), published between database inception and Sept 12, 2025. No restrictions were applied regarding language, study design, or publication date. The search terms were: “breast cancer” [Title/Abstract] AND “brain metastases” [Title/Abstract] AND “ARX788” [Title/Abstract]. This search did not identify any clinical trials evaluating ARX788 therapy for HER2-positive breast cancer and BrMs. We also searched ClinicalTrials.gov using the terms “breast cancer,” “ARX788,” and “Phase 1, 2, 3,” which yielded 12 clinical trials. However, none of these trials specifically evaluated the efficacy of ARX788 in patients with HER2-positive breast cancer plus BrMs. We aimed to address this knowledge gap.

**Added value of this study**

To our knowledge, this prospective, single-arm phase 2 trial is the first study to investigate ARX788 in patients with HER2-positive breast cancer and BrMs. Whilst acknowledging the small sample size (n = 32) and the limitations of this study design, our findings demonstrated the intracranial efficacy of ARX788 for active BrMs in HER2-positive breast cancer patients previously treated with trastuzumab, taxanes, and tyrosine kinase inhibitor (TKI)-containing treatments. The central nervous system (CNS) clinical benefit rate (CBR) was 34.4%, (95% CI: 18.6–53.2; 11 of 32 patients) and the proportion of patients with confirmed CNS overall response rate (ORR) was 25.0% (95% CI: 11.5–43.4). Median CNS progression-free survival (PFS) was 5.6 (95% CI: 3.0–7.4) months. The most frequent grade ≥3 treatment-related adverse events were blurred vision (9.4%), interstitial lung disease/pneumonitis (6.3%), keratopathy (6.3%), dry eyes (3.1%), and thrombocytopaenia (3.1%). The rate of grade ≥3 gastrointestinal toxicity was less than 1%. Therefore, ocular events were the most common adverse events but were generally manageable, while haematological and gastrointestinal toxicities remained infrequent. Interstitial lung disease/pneumonitis requires careful clinical monitoring given its potential severity.

**Implications of all the available evidence**

Our findings on the safety and efficacy of ARX788 show support for further investigation into the use of ARX788 for patients with HER2-positive breast cancer and active BrMs, and offer valuable clinical insights into the management of treatment-related adverse events. Further investigation in larger cohorts, with extended longitudinal follow-up, is warranted.

## Introduction

Human epidermal growth factor receptor (HER) 2–positive breast cancer represents 15–20% of all breast cancers,^1^^,^^2^ and up to 30–50% of patients with advanced or metastatic HER2-positive breast cancer will develop brain metastases (BrMs).^3^^,^^4^ Although local treatments, including stereotactic radiotherapy, whole-brain radiation therapy (WBRT), and surgical resection, are standard therapy for BrMs,^5^ intracranial progression often recurs within 6–12 months,^6^^,^^7^ likely due to undetectable micrometastases beyond the reach of local therapy. Therefore, additional systemic treatment options for patients with BrMs are needed, especially those with active (untreated or previously treated but progressing) BrMs.

Currently, the intracranial activity with small-molecule tyrosine kinase inhibitor (TKI) is well established. The HER2CLIMB trial demonstrated that the combination of tucatinib, capecitabine, and trastuzumab achieved a central nervous system overall response rate (CNS-ORR) of 47.3% and a median CNS progression-free survival (PFS) of 9.6 months in patients with active BrMs,^8^ establishing this triplet as an effective systemic therapy for HER2-positive breast cancer patients with active BrMs after prior treatment. Intracranial activity has also been observed with neratinib plus T-DM1, with 38.1%–50.0% of patients with HER2-positive active BrMs experiencing either stable disease at six months or an objective response.^9^ And the intracranial objective response rate of pyrotinib plus capecitabine was 42.1% for patients who had radiotherapy-treated HER2-positive BrMs.^10^ However, the efficacy of antibody–drug conjugates (ADC) in patients with active BrMs remains to be fully elucidated. The pooled analysis of DESTINY-Breast01, 02, and 03 demonstrated that T-DXd achieved a CNS-ORR of 45.5% in patients with HER2-positive active BrMs.^11^ Similarly, the phase 2 DEBBRAH study reported an intracranial ORR of 46.2% (95% CI: 19.2–74.9) in this population.^12^ In the DESTINY-Breast12 study, T-DXd achieved a CNS-ORR of 62.3% in patients with HER2-positive active BrMs,^13^ establishing it as a preferred therapy. But its relatively high rates of gastrointestinal and haematologic toxicity^13^ underscore the need to develop new ADC therapies.

ARX788 is a next-generation ADC in which an anti-HER2 monoclonal antibody is site-specifically linked via the unnatural amino acid para-acetylphenylalanine (pAF) to Amberstatin269 (AS269), a proprietary version of monomethyl auristatin F (MMAF) payload. Employing the EuCODE™ platform to form a highly stable oxime bond yields a homogeneous construct with a drug-to-antibody ratio of approximately two.^14^ In the randomised phase 3 ACE-Breast-02 trial, ARX788 significantly prolonged PFS in HER2-positive advanced breast cancer (11.3 months vs. 8.2 months, HR = 0.64, P = 0.0006) when compared with lapatinib plus capecitabine, underscoring its therapeutic potential.^15^ However, patients with active BrMs were excluded from the ACE-Breast-02 trial. Here, we report results from the prospective phase 2 ACE-Breast-06 study (NCT05018702), a non-comparative study that evaluated the efficacy and safety of ARX788 in patients with HER2-positive breast cancer and active BrMs.

## Methods

### Study design, participants, and ethics

This study is a prospective, single-arm, single-centre, phase 2 study involving patients with pathologically confirmed HER2-positive metastatic breast cancer. HER2-positive was defined as immunohistochemistry (IHC) 3+ or fluorescence in situ hybridisation (FISH)-positive. Patients were included if they were aged 18–75, had received trastuzumab, taxane, and TKI-containing treatment, and had at least one MRI-confirmed ≥1 cm (maximum diameter) brain metastatic lesion, that were newly diagnosed untreated BrMs, or BrMs that had progressed since prior local therapies but had not been re-treated with local interventions after progression. Mannitol, bevacizumab, or glucocorticoids therapy were allowed, only if the dose has been stable for ≥1 week prior to the study entry. In addition, patients should have an Eastern Cooperative Oncology Group (ECOG) score of 0–2, an absolute neutrophil count of ≥1.5 × 10^9^/L, a platelet count of ≥75 × 10^9^/L, a haemoglobin count of ≥90 g/L, an international normalised ratio (INR) of ≤1.5, an activated partial thromboplastin time (APTT) of ≤1.5 × ULN, a creatinine clearance (calculated by Cockcroft-Gault formula) of ≥50 ml/min or a serum creatinine of ≤1.5 × ULN, an aspartate aminotransferase (AST)/alanine aminotransferase (ALT) of ≤2.5 × ULN (or ≤5 × ULN for patients having liver metastases), a left ventricular ejection fraction (LVEF) ≥50%, and a QT interval corrected by Fridericia's formula (QTcF) < 470 ms (female) or <450 ms (male). All prior therapy-related adverse events must have recovered to ≤ grade 1, with the exception of alopecia, hyperpigmentation, irreversible late effects of radiotherapy, and platinum induced grade 2 neurotoxicity.

Patients were excluded if they had any of the following: leptomeningeal metastases or cystic BrMs confirmed by MRI or cerebrospinal fluid analysis, massive pleural effusion or ascites not controllable by drainage or other interventions, BrMs requiring urgent local therapy, previous treatment with T-DM1 or other anti-HER2 ADC drugs, palliative bone radiotherapy or major surgery within 2 weeks prior to the first dose, received trastuzumab or endocrine therapy within 1 weeks prior to the first dose, history of interstitial lung disease (ILD) or radiation pneumonitis requiring corticosteroid therapy, clinical or radiographic evidence of active ILD, keratitis or corneal diseases or retinal diseases or active ocular infection requiring intervention, receive any other anti-cancer treatment during the study (except low-dose bevacizumab for cerebral edema or bisphosphonates for bone metastases/osteoporosis), and history of another malignancy within 5 years (except adequately treated in situ cervical carcinoma or non-melanoma skin cancer).

This study was undertaken in accordance with the ethical principles originating in the Declaration of Helsinki, the International Conference on Harmonisation (ICH) E6 Guidelines for Good Clinical Practice, and all applicable local regulations. This study was approved (reference 2104234-11) by the ethics committee of the Fudan University Shanghai Cancer Center (Shanghai, China). Written informed consent was obtained from each patient before study participation.

### Procedures

ARX788 was administered intravenously every 21 days at a dose of 1.5 mg per kg of body weight until disease progression, unacceptable toxicity, withdrawal of consent, or other situation determined by the investigator to warrant treatment discontinuation. During the treatment period, tumour response was assessed every 6 weeks. The standard imaging evaluations included contrast-enhanced CT or MRI of the chest, abdomen, and pelvis; contrast-enhanced brain MRI; and a baseline bone emission computed tomography. Additional imaging examinations, such as breast MRI, mammography, breast ultrasound, or site-specific imaging for suspected metastases, could be performed based on the clinical status of each patient. Disease progression was defined as either intracranial progression, based on the Response Assessment in Neuro-Oncology Brain Metastases (RANO-BM) criteria,^16^ and/or extracranial progression, based on the Response Evaluation Criteria in Solid Tumours (RECIST) version 1.1.^17^ If extracranial progression were observed, the decision regarding subsequent therapy was made by the investigator. In cases of intracranial progression only, patients were recommended to continue ARX788 treatment in combination with local therapy. The protocol is available in the Supplementary Materials.

### Randomisation and masking

This single-centre study involved no randomisation. This open-label trial had no masking or allocation concealment.

### Outcomes

The primary endpoint was clinical benefit rate (CBR) in the CNS, which was defined as proportion of patients with complete response (CR)/partial response (PR)/stable disease (SD) at ≥24 weeks by RANO-BM.

Secondary endpoints consisted of CNS PFS (interval between first dose and CNS disease progression or death), PFS (interval between first dose and disease progression or death), OS (interval between first dose and death by any cause), CNS ORR (proportion of patients with confirmed CNS complete or partial response), sites of next progression (extracranial vs CNS vs both), and safety. Efficacy analyses were performed in the full analysis set (FAS; patients who were enrolled in the trial and received at least one dose of ARX788).

### Safety

Safety analyses were conducted based on the safety set (SS; identical to the FAS). All adverse events (AEs) were graded according to the National Cancer Institute Common Terminology Criteria for Adverse Events (NCI-CTCAE) version 5.0. Descriptive statistics were conducted to summarise safety data, including AEs, grade ≥3 AEs, serious adverse events (SAEs), AEs leading to dose modification, and AEs leading to treatment discontinuation.

### Statistical analysis

The sample size was estimated based on the primary endpoint CNS CBR. This study aims to enrol patients with HER2-positive, treatment-refractory breast cancer with active BrMs. As there is a lack of published data on the CNS CBR in this population, it was conservatively estimated at 10%. It is hypothesised that treatment with ARX788 could improve the CNS CBR to 30%. A Simon's two-stage design was employed to calculate the sample size, using a two-sided test with a type I error rate of 5% and a power of 80%. In the first stage, 10 patients will be enrolled. If ≤ 1 patient achieves CR/PR/SD ≥ 24 weeks, the study will be terminated. Otherwise, the study will proceed to enrol a total of 29 patients. If ≤ 5 patients achieve CR/PR/SD ≥ 24 weeks among the 29 patients, the treatment will be considered ineffective; otherwise, the treatment will be deemed effective. Assuming a 10% dropout rate, a total of 32 patients should be enrolled. PFS, CNS PFS, OS were analysed by the Kaplan–Meier method. ORR was measured from the first dose until the disease progression or, if no progression occurred, until the last evaluable assessment—regardless of treatment discontinuation. CNS ORR was measured from the first dose until the CNS disease progression or, if no progression occurred, until the last evaluable assessment—regardless of treatment discontinuation. For PFS, patients without disease progression or death at the time of analysis were censored at their date of last evaluable assessment. For CNS PFS, patients without CNS disease progression or death at the time of analysis were censored at their date of last evaluable assessment. The two-sided 95% confidence intervals such as CNS CBR were calculated using the Clopper–Pearson exact method. No sensitivity or post-hoc analyses were performed. Data analyses were completed using prism (version 9.0).

### Role of the funding source

The study funders were not involved in the study design, data collection, data analyses, data interpretation, or writing of report.

## Results

### Patients and baseline characteristics

**Fig. 1:**
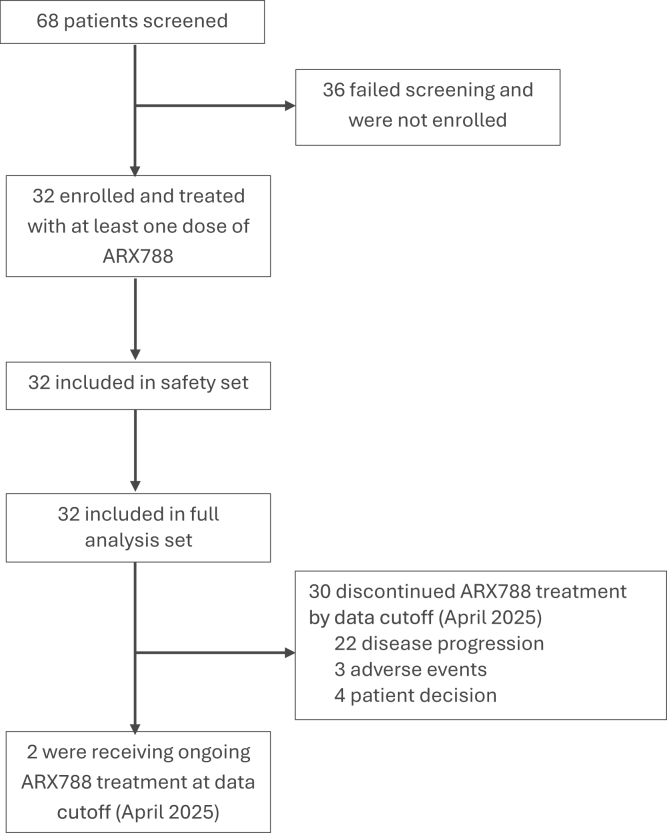
Flowchart of the study.

Between August 2021 and April 2025, 32 patients were included and treated with at least one dose of ARX788 (Fig. 1). The median follow-up duration was 15.0 months (range, 1.8–42.5), and two patients were continuing to receive ARX788 treatment at final data cutoff (April 2025). The most common reasons for treatment discontinuation were disease progression (22/30, 73.3%), patients’ choice (4/30, 13.3%), and adverse event (3/30, 10%).

All the included patients had active HER2-positive BrMs with no clinical indication for immediate local treatment. The baseline clinical and demographic characteristics of the cohort were summarised in Table 1. 25.0% of patients received ARX788 as second line treatment in the metastatic setting, 40.6% as third line therapy, and the remaining patients as fourth-line or later therapy. 13 (40.6%) patients received prior CNS radiotherapy and 2 (6.3%) patients received prior brain surgery. All patients had previously received trastuzumab and taxane, with 13 (40.6%) having received pertuzumab. All the patients had been treated with TKI, including pyrotinib (90.6%) and lapatinib (9.4%).

**Table 1:** Demographics and baseline clinical characteristics.

	Patients (n = 32)
Age, median (range), years	51 (34–67)
Sex	
Female	32 (100%)
Histology, WHO classification, n (%)	
Ductal	30 (93.8%)
Others	2 (6.2%)
Hormone receptor	
ER- or PR-positive	14 (43.8%)
ER- and PR-negative	18 (56.2%)
Metastatic sites at baseline excluding brain metastases, n (%)	
Liver metastases	7 (21.9%)
Lung metastases	19 (59.4%)
Bone metastases	15 (46.9%)
Measurable disease at baseline	
Yes	32 (100%)
Lines in the metastatic setting, n (%)	
2	8 (25.0%)
3	13 (40.6%)
4	4 (12.5%)
≥5	7 (21.9%)
Prior HER2 inhibitor agents, n (%)	
Trastuzumab	32 (100%)
Pertuzumab	13 (40.6%)
Lapatinib	3 (9.4%)
Pyrotinib	29 (90.6%)
Prior CNS therapies, n (%)	
Brain surgery	2 (6.3%)
CNS radiotherapy	13 (40.6%)

### Antitumour efficacy

**Fig. 2:**
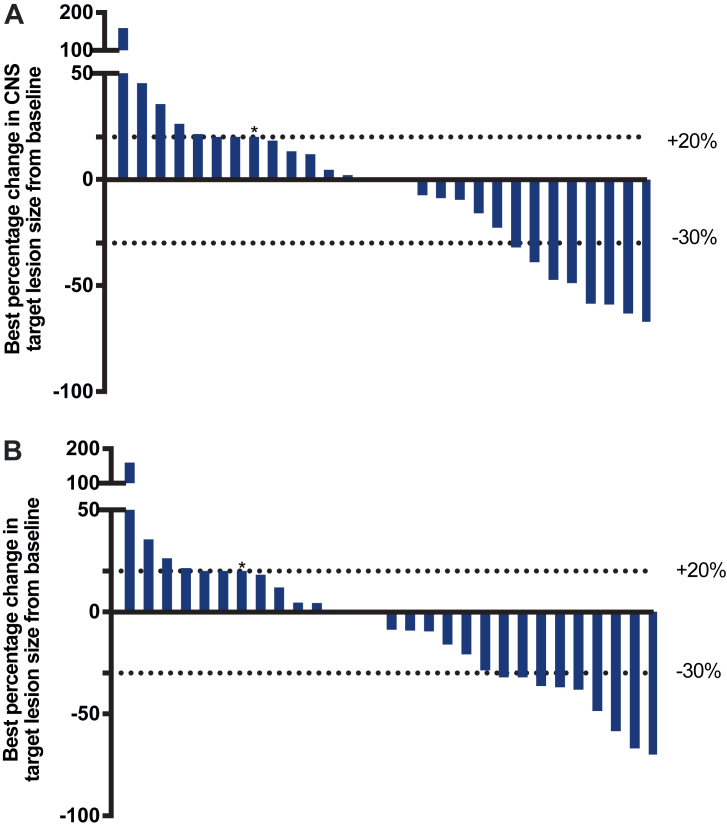
**(A)** Best percentage change from baseline in CNS target lesions size. (**B**) Best percentage change from baseline in target lesions size. Analyses were performed in 32 patients, except for three who had no post-baseline evaluation. If a patient developed a new lesion or showed progression of non-target lesions, a +20% change from baseline was imputed as their best response. The lower dashed line denotes a 30% decrease in tumour size, corresponding to a partial response. The upper dashed line denotes a 20% increase in tumour size, corresponding to a progressive disease. Abbreviations: CNS, central nervous system.

**Fig. 3:**
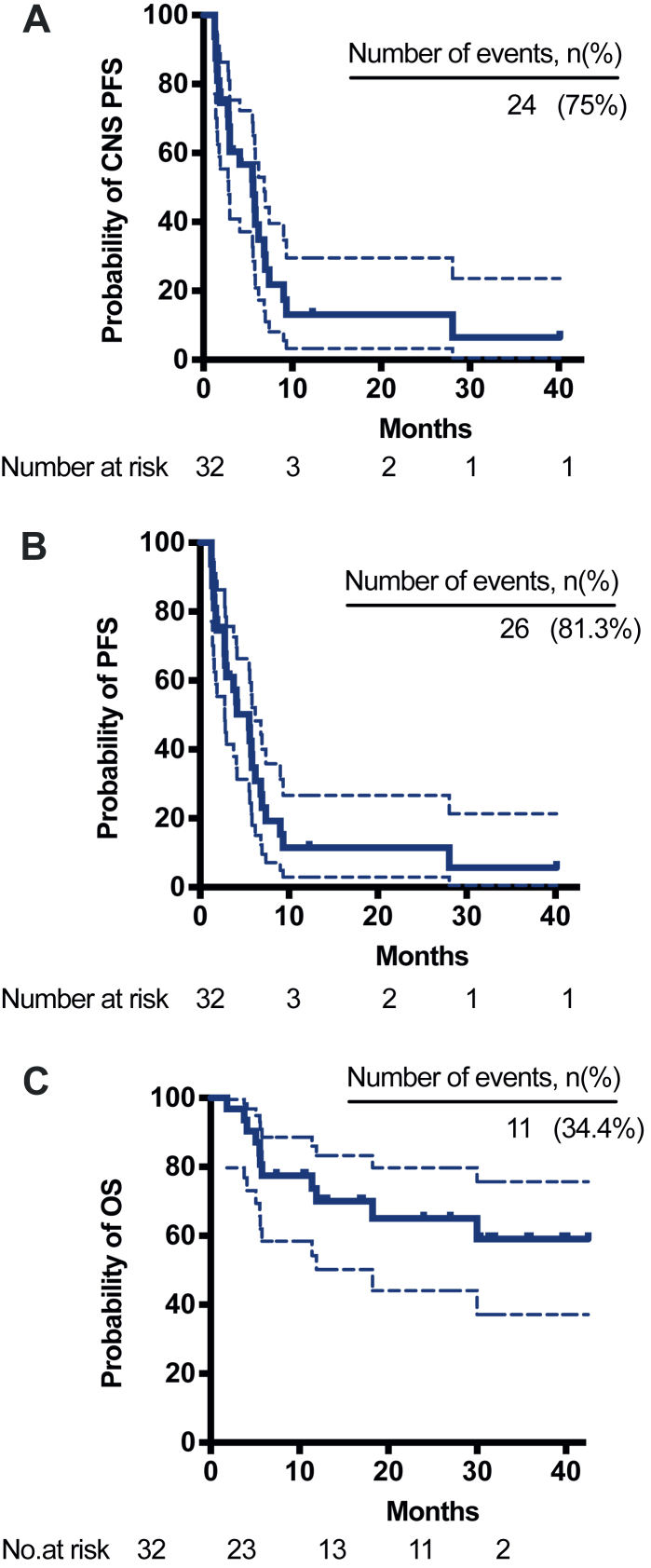
(**A**) Kaplan–Meier analysis of CNS PFS. (**B**) Kaplan–Meier analysis of overall PFS. (**C**) Kaplan–Meier analysis of OS. CNS PFS was defined as interval between first dose and CNS disease progression or death. Overall PFS was defined as interval between first dose and disease progression or death. OS was defined as interval between first dose and death by any cause. Abbreviations: PFS, Progression; OS, overall survival; CNS, central nervous system.

**Fig. 4:**
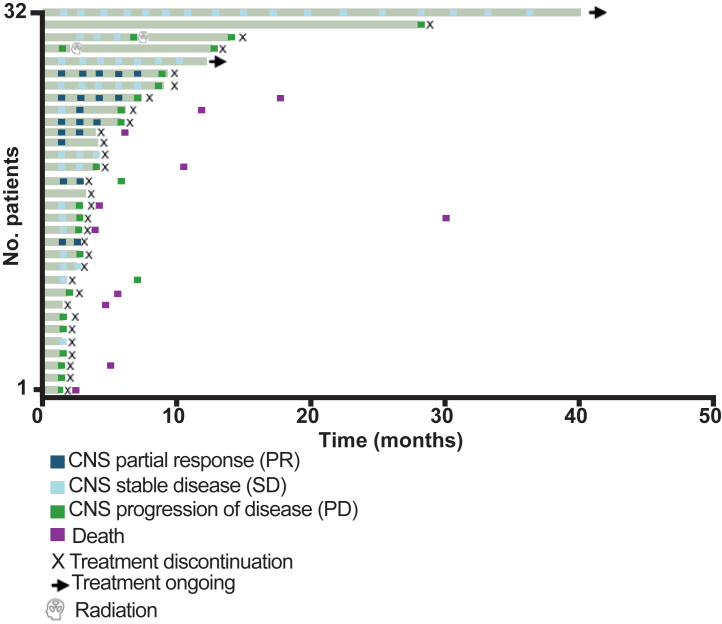
The CNS objective response and durations of treatment of the 32 patients are summarised in the swimmer plot, with two patients continuing to receive ARX788 treatment at the data cutoff. Abbreviations: CNS, central nervous system.

The best percentage change in target lesions were shown in Fig. 2. As for the CNS efficacy, the 6-month CNS PFS was 39.3% (95% CI: 20.9–57.2) and the median CNS PFS was 5.6 (95% CI: 3.0–7.4) months (Table 2 and Fig. 3A). A total of 8 (25.0%) patients had a PR, and 3 (9.4%) patients had a SD ≥ 24 weeks. The proportion of patients with confirmed CNS ORR was 25.0% (95% CI: 11.5–43.4), and CNS CBR was reported in 11 out of 32 patients (34.4%, 95% CI: 18.6–53.2). As for the overall efficacy, the 6-month PFS was 34.8% (95% CI: 18.1–52.1) and the median PFS was 5.5 (95% CI: 2.8–7.0) months (Table 2 and Fig. 3B). The OS at 12 months was 70.1% (95% CI: 50.3–83.3) and the median OS data was immature at the time of analyses (Table 2 and Fig. 3C). Among the 24 patients with disease progression, 2 (8.3%) experienced extracranial progression alone, 17 (70.8%) experienced intracranial progression alone, and 5 (20.8%) experienced both intracranial and extracranial progression (Table 3). Among those with intracranial progression alone, two patients continued ARX788 after receiving whole-brain radiotherapy (Fig. 4). Notably, the intervals between the first and second intracranial progression were 7 months and 11 months, respectively.

**Table 2:** Overall anti-tumour activity.

	Patients (N = 32)
6-month PFS, % (95% CI)	34.8% (95% CI: 18.1–52.1)
6-month CNS PFS, % (95% CI)	39.3% (95% CI: 20.9–57.2)
12-month OS, % (95% CI)	70.1 (50.3–83.3)
Median PFS, months (95% CI)	5.5 (2.8–7.0)
Median CNS PFS, months (95% CI)	5.6 (3.0–7.4)
Median OS, months (95% CI)	NR
Best CNS objective response, n (%)	
Complete response	0 (0.0)
Partial response	8 (25.0)
Stable disease	13 (40.6)
Stable disease ≥24 weeks	3 (9.4)
Progressive disease	8 (25.0)
Not evaluable	3 (9.4)
Confirmed CNS ORR, % (95% CI)	25.0 (11.5–43.4)
Confirmed CNS CBR, % (95% CI)	34.4 (18.6–53.2)

**Table 3:** Site of first progression.

The site of first progression	Patients with disease progression (N = 24)
Extracranial alone	2 (8.3%)
Intracranial alone	17 (70.8%)
Both intracranial and extracranial	5 (20.8%)

Among the 30 patients who discontinued ARX788 treatment, 9 patients (30.0%) received T-DXd, 1 patient (3.3%) received T-DM1, and 1 patient (3.3%) received another anti-HER2 ADC. In addition, 5 patients (16.7%) received anti-HER2 TKI–based regimens, and 3 patients (10.0%) received anti-HER2 TKI plus trastuzumab–based regimens. 7 patients (23.3%) did not receive any further systemic therapy from progression until death, and post-progression treatment information was unavailable for 4 patients (13.3%).

### Safety

The median treatment cycle was 4 (range, 1–44). The most common treatment-related adverse events (TRAEs), at any grade and grade ≥3, are shown in Table 4. TRAEs at any grade were observed in 31 (96.9%) of 32 patients. Grade ≥3 TRAEs occurred in four (12.5%) of 32 patients (Table 4). TRAEs mainly included increased ALT/AST, hypertriglyceridemia, haematuria, hypokalemia, and hypercholesterolaemia. The most frequent grade ≥3 TRAEs were blurred vision (9.4%), keratopathy (6.3%), dry eyes (3.1%), thrombocytopaenia (3.1%), and interstitial lung disease (3.1%). The rate of grade ≥3 haematological toxicity was less than 4%, and that of grade ≥3 gastrointestinal toxicity was less than 1%. Three (9.4%) patients discontinued ARX788 treatments due to TRAE: two due to ILD, and one due to pneumonitis.

**Table 4:** Most common treatment-related adverse events (≥10%) among the 32 patients.

	All grades, n (%)	Grade ≥3, n (%)
Any adverse events	31 (96.9%)	4 (12.5%)
Haematological toxicities		
Anaemia	8 (25.0%)	0
Thrombocytopaenia	6 (18.8%)	1 (3.1%)
Hepatotoxity		
AST increased	16 (50.0%)	0
ALT increased	10 (31.2%)	0
Elevated alkaline phosphatase	6 (18.8%)	0
Elevated total bilirubin	3 (9.4%)	0
Ocular toxicity		
Dry eyes	9 (28.1%)	1 (3.1%)
Keratopathy	7 (21.9%)	2 (6.3%)
Blurred vision	6 (18.8%)	3 (9.4%)
Respiratory toxicity		
Interstitial pneumonitis	3 (9.4%)	1 (3.1%)
Renal toxicity		
Haematuria	12 (37.5%)	0
Proteinuria	6 (18.8%)	0
Other toxicity		
Hypertriglyceridemia	13 (40.6%)	0
Hypokalemia	9 (28.1%)	0
Hypercholesterolaemia	9 (28.1%)	0
Hyperuricemia	6 (18.8%)	0
Hyponatremia	5 (15.6%)	0
Hypoalbuminemia	4 (12.5%)	0
Hypercalcaemia	4 (12.5%)	0
Hyperglycemia	4 (12.5%)	0
Alopecia	4 (12.5%)	0

ILD occurred in three (9.4%) patients, predominantly grade1/2 (6.3%), with one grade ≥3 event (3.1%). ARX788 treatment was discontinued in two cases due to ILD, but no ILD-related deaths were reported. The median onset time of ILD was 128 days. When combined with pneumonitis, the overall incidence of ILD/pneumonitis was 21.9% (7/32 patients), including 15.6% (5/32) with grade 1/2 events and 6.3% (2/32) with grade ≥3 events. A grade 5 ILD/pneumonitis event occurred in one (3.1%) patient.

Ocular toxicity related to ARX788 included blurred vision (18.8%), dry eye (28.1%), and keratopathy (21.9%). No cases of ulceration, perforation, or blindness were observed. No grade 4 events occurred. All ocular toxicities were reversible following dose reduction, and none led to treatment discontinuation.

## Discussion

To our knowledge, the ACE-Breast-06 study is the first prospective trial to report the intracranial activity of ARX788. This study demonstrated efficacy in HER2-positive breast cancer patients previously treated with trastuzumab, taxanes, and TKI-containing treatments, achieving a CNS CBR of 34.4%, a CNS ORR of 25.0%, and a median CNS PFS of 5.6 months.

Initial reports indicated that trastuzumab has minimal activity in the CNS due to its inability to effectively cross the blood–brain barrier. Consequently, researches on HER2-positive breast cancer BrMs has focused on small molecule TKIs, including lapatinib,^18^ tucatinib,^8^ pyrotinib and neratinib.^19^ In patients with treatment-naïve HER2-positive BrMs, lapatinib plus capecitabine achieved an ORR of 66%.^20^ However, in a cohort of heavily pretreated patients, the ORR of lapatinib plus capecitabine dropped to 21%.^21^ Therefore, evaluating the efficacy of ARX788 in TKI-pretreated patients is of critical clinical importance. Recent studies indicated that anti-HER2 ADC exhibit promising efficiency against HER2-positive BrMs. In preclinical mouse models of HER2-positive breast cancer BrMs, T-DM1, but not trastuzumab, significantly delayed tumour growth by inducing apoptosis and mitotic catastrophe despite similar tissue distribution.^22^ In the post hoc exploratory analysis of patients with HER2-positive BrMs enrolled in the KAMILLA trial, T-DM1 achieved an intracranial ORR of 21.4% (95% CI: 14.6–29.6%).^23^ Our reported ORR of ARX788 is within the range of those described with T-DM1, while T-DXd yielded superior CNS ORR in the DESTINY-Breast12 in patients with HER2-positive active BrMs (CNS ORR 62.3% [95% CI: 50.1%–74.5%]).^13^ Notably, 59.5% of KAMILLA participants had received prior anti-HER2 TKI therapy, compared with only ∼5% in DESTINY-Breast12 and 100% in our cohort. Consequently, the lower ORR observed with ARX788 vs T-DXd may, at least in part, caused by our study's predominance of TKI-pretreated patients. However, cross-trial comparisons must be interpreted with caution and regarded as hypothesis-generating. Moreover, our study did not include stable BrMs, because the randomised phase 3 ACE-Breast-02 trial had already included those population and its subgroup analysis indicated a favourable trend in OS. Therefore, the ACE-Breast-06 trial instead enrolled patients with active BrMs, who were excluded from ACE-Breast-02, to specifically assess the efficacy of ARX788 in this high–unmet-need population.

ARX788 was generally well tolerated, with a TRAE-related discontinuation rate of only 9.4%. Grade ≥3 adverse events were predominantly ocular toxicities and pulmonary toxicities. Notably, ARX788 exhibits low rates of haematological and gastrointestinal toxicity, comparably lower than those reported for T-DXd, although cross-trial comparisons should be interpreted with caution due to potential bias. Ocular toxicity—manifesting as dry eye, blurred vision, and keratopathy—was effectively managed with frequent use of ocular lubricants such as sodium hyaluronate, eyedrops containing calf serum, and other interventions as recommended by ophthalmologists. Therefore, no treatment discontinuation was caused by ocular toxicity. Moreover, in our study, prophylactic calf-serum eye drops were administered to prevent ocular discomfort—a measure not used in the ACE-Breast-02 trial—which was associated with a significantly lower incidence of dry eye (9/32 vs. 121/220; P = 0.007), although reductions in blurred vision (6/32 vs. 77/220; P > 0.05) and keratopathy (7/32 vs. 62/220; P > 0.05) did not reach statistical significance. For future ARX788 treatment, we recommend implementing primary ocular prophylaxis and ensuring early ophthalmology involvement to further improve patients' quality of life.

Pulmonary toxicity was another common adverse event. The overall incidence of ILD/pneumonitis in our cohort was 21.9% (all grades) and 3.1% (grade 5), comparable to the DESTINY-Breast12 trial's findings in patients with BrMs (16% at all grades and 2% at grade 5). Our observed ILD rate for ARX788 was 9.4% (all grades) and 3.1% (≥ grade 3), which is numerically higher than the DESTINY-Breast12 results (6.5% and 1.1%, respectively). The higher incidence of ILD observed with ARX788 may be attributable to several factors, including the enrolment of patients with pre-existing chronic pulmonary conditions, the protocol-mandated chest CT scans performed every six weeks, heightened clinical vigilance and awareness of drug-related pulmonary toxicities, and the smaller sample size of our study. Notably, in the DESTINY-Breast12 trial, protocol guidelines mandated permanent discontinuation of T-DXd in any patient who developed grade 2 ILD. In contrast, our study's protocol allowed investigators to pause or reduce the ARX788 dose for grade 2 ILD, with the option to resume treatment if the event resolved to ≤ grade 1 within 12 weeks. Of the 3 patients who developed ILD in our trial, all initially presented with grade 2 ILD: one patient's ILD improved to ≤grade 1 within 8 weeks following a dose reduction to 1.3 mg/kg. The remaining two patients permanently discontinued ARX788—one because ILD persisted beyond 12 weeks and the other due to progression to grade 4. These findings suggest that what specific criteria should be met before re-challenge ARX788 after temporary interruption in patients with initial grade 2 ILD remains uncertain and warrants validation in larger cohorts. Moreover, ARX788-related ILD was a late-onset toxicity, typically emerging at a median of 128 days after the initial dose, and therefore demands vigilant long-term monitoring. Our recommendations included patient and family education; regular fingertip pulse oximetry to detect SpO_2_ < 95% at any time or place; and proactive management, combining steroid therapy with anti-infective agents whenever clinical signs suggested infection.

This study has several limitations. First, its open-label, single-arm design, coupled with the lack of independent review of responses. Second, the marked differences in baseline characteristics between our cohort and relevant historical comparator cohorts (such as rates of prior anti-HER2 TKI therapy, number of previous treatment lines, and primary endpoints) makes cross-trial comparisons challenging. Third, prior intracranial radiotherapy in some patients complicated BrMs lesion assessment, as radiation necrosis can be difficult to distinguish from true metastatic lesions. Fourth, this study excluded patients previously treated with anti-HER2 ADCs, which may limit the generalisability of our findings to patients pretreated with anti-HER2 ADCs. Finally, quality of life was not assessed in this study. Future prospective studies incorporating patient-reported outcomes could better capture the clinical benefit of ARX788.

In conclusion, ARX788, a site-specific homogeneous ADC, demonstrated intracranial efficacy for active BrMs in HER2-positive breast cancer patients previously treated with trastuzumab, taxanes, and TKI-containing treatments. Ocular events were the common adverse events but were generally manageable, while haematologic and gastrointestinal toxicities remained infrequent. ILD/pneumonitis requires careful clinical monitoring given its potential severity. These findings support ARX788 as a potential treatment alternative for patients with HER2-positive active BrMs.

## Contributors

JZ and XCH share corresponding author, who accessed and verified the underlying data. TL and MXL are first authors who responsible for the statistical analysis of the research data and the drafting of the manuscript. All authors participated in data acquisition, analysis, or interpretation. All authors were responsible for the decision to submit the manuscript. All authors participated in review of the manuscript.

## Data sharing statement

Any additional information required to reanalyse the data reported in this paper is available from the corresponding author Jian Zhang (jianz@fudan.edu.cn) upon reasonable request.

## Declaration of interests

We declare no competing interests.

## Appendix A Supplementary data

Study protocol
