# Supplementary material for: Efficacy and safety of ARX788 for individuals with HER2-positive breast cancer and brain metastases (ACE-Breast-06): a single-arm, phase 2 trial in China
Source: eClinicalMedicine. 2025 Oct 30;90:103614. doi: 10.1016/j.eclinm.2025.103614 (PMC12615312; doi:10.1016/j.eclinm.2025.103614)
Supplement: Study protocol [file mmc1.docx]

**Study protocol**

**1. Brief Summary**

A Phase 2 Study of ARX788 in HER2-positive, Metastatic Breast Cancer Patients whose Disease is resistant or refractory to Tyrosine kinase inhibitors (TKI).

**2. Detailed Description**

This is an open-label, single arm, phase 2 study of ARX788 in HER2-positive, metastatic breast cancer patients whose disease is resistant or refractory to TKI. The ARX788 will be administered every 3 weeks (Q3W) intravenous (IV) infusion. In this study, Simon's two-stage design is used. Subjects received treatment until disease progression, intolerable toxicity, withdrawal from the study, or discontinuation judged by the investigator. Drug efficacy and safety data will be collected.

**3. Official Title**

A Prospective, Single-arm, Single-center Phase II Clinical Study of Recombinant Humanized Anti-HER2 Monoclonal Antibody-AS269 Conjugate (ARX788) in the Treatment of HER2-positive Breast Cancer Patients With Brain Metastases

**3. Conditions**

HER2-positive, Metastatic Breast Cancer

**4. Intervention / Treatment**

Drug: ARX788

**5.Study ID Numbers**

ACE-Breast-06

**6.Inclusion Criteria:**

- Age ≥18 years, and ≤75 years, male or female;
- Eastern Cooperative Oncology Group (ECOG) performance status of 0~2;
- Breast cancer patients diagnosed as HER2-positive by pathological examination;
- Metastatic breast cancer subjects previously treated with trastuzumab, taxane and TKI-containing regimens;
- Had at least one MRI-confirmed ≥1cm (maximum diameter) brain metastatic lesion, that were newly diagnosed untreated brain metastases, or brain metastases that had progressed since prior local therapies but had not been re-treated with local interventions after progression;
- Mannitol, bevacizumab, or glucocorticoids therapy were allowed, only if the dose has been stable for ≥1 week prior to the study entry;
- Adequate organ functions;
- Acute toxicities from any prior therapy, surgery, or radiotherapy must have resolved to Grade ≤1;
- Patients who participate in the trial voluntarily, sign an informed consent, have good compliance and are willing to comply with the follow-up visit.

**7. Exclusion Criteria:**

- Leptomeningeal metastases or cystic metastases confirmed by MRI or lumbar puncture;
- Massive pleural effusion or ascites not controllable by drainage or other interventions;
- Brain metastases requiring urgent local therapy;
- Previous treatment with T-DM1 or other anti-HER2 ADC drugs;
- Received a whole-brain radiotherapy, chemotherapy, or surgery within 2 weeks prior to the first dose of ARX788, or trastuzumab-targeted therapy or endocrine therapy within 1 week, or palliative radiotherapy for bone metastases within 2 weeks;
- History of interstitial lung disease (ILD) or radiation pneumonitis requiring corticosteroid therapy, clinical or radiographic evidence of active ILD
- Keratitis, corneal diseases, retinal diseases or active eye infections that require intervention;
- Unwilling or unable to stop wearing contact lenses for the duration of the study;
- Participated in other clinical trials within 2 weeks prior to enrollment;
- Receiving any antitumor therapy for any other tumor, bevacizumab for the control of brain edema and bisphosphonates for the treatment of bone metastases or the prevention of osteoporosis are the exception;
- With a history of any malignancies other than breast cancer in the past 5 years, excluding cured cervical carcinoma in situ, basal cell carcinoma of the skin or squamous cell carcinoma of the skin;
- Cardiac insufficiency;
- Uncontrolled hypertension;
- History of allergic reactions to any component of ARX788, or with a history of protein drug allergy, a history of specific allergies (asthma, rheumatism, eczematous dermatitis), or a history of other severe allergic reactions, who are unsuitable for ARX788 treatment as per the investigator's judgments;
- Pregnancy or lactation;
- History of immunodeficiency, including HIV-positive, or other acquired or congenital immunodeficiency diseases, or a history of organ transplantation;
- Current known active infection with human immunodeficiency virus (HIV), hepatitis B virus, hepatitis C virus or syphilis;
- History of neurological or psychiatric disorder, including epilepsy or dementia;
- Suffering severe or uncontrolled systemic diseases.

**8. Study design and treatment**

- ARX788 was administered intravenously every 21 days at a dose of 1.5 mg per kg of body weight until disease progression, unacceptable toxicity, withdrawal of consent, or other situation determined by the investigator to warrant treatment discontinuation.
- During the treatment period, tumor response was assessed every 6 weeks.
- The standard imaging evaluations included contrast-enhanced CT or MRI of the chest, abdomen, and pelvis; contrast-enhanced brain MRI; and a baseline bone emission computed tomography
- Additional imaging examinations, such as breast MRI, mammography, breast ultrasound, or site-specific imaging for suspected metastases, could be performed based on the clinical status of each individual patient.
- Disease progression was defined as either intracranial progression, based on the Response Assessment in Neuro-Oncology Brain Metastases (RANO-BM) criteria^14^, and/or extracranial progression, based on the Response Evaluation Criteria in Solid Tumors (RECIST) version 1.1^15^.
- If extracranial progression were observed, the decision regarding subsequent therapy was made by the investigator.
- In cases of intracranial progression only, patients were recommended to continue ARX788 treatment in combination with local therapy.

9. **Endpoints**

- The primary endpoint was clinical benefit rate (CBR) in the CNS, which was defined as proportion of patients with complete response (CR)/partial response (PR)/stable disease (SD)≥24 weeks by RANO-BM.
- Secondary endpoints consisted of CNS PFS (interval between first dose and CNS disease progression or death), PFS (interval between first dose and disease progression or death), OS (interval between first dose and death by any cause), CNS ORR (proportion of patients with confirmed CNS complete or partial response), sites of next progression (extracranial versus CNS versus both), and safety.
- Efficacy analyses were performed in the full analysis set (FAS; patients who were enrolled in the trial and received at least one dose of ARX788).

**10. Safety**

- Safety analyses were conducted based on the safety set (SS; identical to the FAS).
- All adverse events (AEs) were graded according to the National Cancer Institute Common Terminology Criteria for Adverse Events (NCI-CTCAE) version 5.0.
- Descriptive statistics were conducted to summarize safety data, including AEs, grade ≥3 AEs, serious adverse events (SAEs), AEs leading to dose modification, and AEs leading to treatment discontinuation.

**11. Statistical analysis**

- The sample size was estimated based on the primary endpoint CNS CBR. This study aims to enroll patients with HER2-positive, treatment-refractory breast cancer with active brain metastases. As there is a lack of published data on the CNS CBR in this population, it was conservatively estimated at 10%. It is hypothesized that treatment with ARX788 could improve the CNS CBR to 30%. A Simon’s two-stage design was employed to calculate the sample size, using a two-sided test with a type I error rate of 5% and a power of 80%. In the first stage, 10 patients will be enrolled. If ≤1 patient achieves CR/PR/SD≥24, the study will be terminated. Otherwise, the study will proceed to enroll a total of 29 patients. If ≤5 patients achieve CR/PR/SD ≥24 weeks among the 29, the treatment will be considered ineffective; otherwise, the treatment will be deemed effective. Assuming a 10% dropout rate, a total of 32 patients should be enrolled.
- PFS, CNS PFS, OS were analyzed by the Kaplan-Meier method. ORR was measured from the first dose until the disease progression or, if no progression occurred, until the last evaluable assessment—regardless of treatment discontinuation.
- CNS ORR was measured from the first dose until the CNS disease progression or, if no progression occurred, until the last evaluable assessment—regardless of treatment discontinuation.
- For PFS, patients without disease progression or death at the time of analysis were censored at their date of last evaluable assessment.
- For CNS PFS, patients without CNS disease progression or death at the time of analysis were censored at their date of last evaluable assessment.
- Data analyses were completed using prism 9.

**研究方案**

（版本号：1.1 版本日期：2021.05.19）

**研究名称：**重组人源化抗HER2单抗-AS269偶联药物（ARX788）治疗HER2阳性乳腺癌伴脑转移患者的前瞻性、单臂、单中心II期临床研究

研究编号：BCBM-002/ACE-Breast-06

**申办单位：**复旦大学附属肿瘤医院

**承担科室：**肿瘤内科

**主要研究者：**胡夕春、张剑

**临床协调人：**李婷

**签字页**

**研究者签字**

本人作为该研究项目的主要负责人，将遵循卫生部《涉及人的生物医学研究伦理审查办法》（2016）、WMA《赫尔辛基宣言》（2013）和CIOMS《人体生物医学研究国际道德指南》（2002）和GCP的伦理原则，在药物临床试验质量管理规范指导下，使用伦理委员会批准的方案，根据本方案要求进行研究，以保证研究的科学性并保护受试者的健康与权利。

**研究单位：复旦大学附属肿瘤医院**

**主要研究者（印刷体） 主要研究者（签名） 签字日期（年/月/日）**

**目 录**

[方案摘要 1](#_Toc63595073)

[临床试验流程图 5](#_Toc63595074)

[缩略语表 8](#_Toc63595075)

[1 研究背景 11](#_Toc63595076)

[2 研究目的及终点指标 11](#_Toc63595077)

[3 研究设计 12](#_Toc63595078)

[4 受试者的选择和退出 13](#_Toc63595079)

[4.1 入组标准 13](#_Toc63595080)

[4.2 排除标准 13](#_Toc63595081)

[4.3 退出标准 15](#_Toc63595082)

[4.4 终止标准 15](#_Toc63595083)

[5 治疗药物 15](#_Toc63595084)

[5.1 研究药物名称和来源 15](#_Toc63595085)

[5.2 ARX788的给药方式 16](#_Toc63595086)

[5.3 ARX788的暂停和剂量下调 16](#_Toc63595087)

[5.4 给药延迟 17](#_Toc63595088)

[5.5 试验药物的管理 18](#_Toc63595089)

[6 合并用药 18](#_Toc63595090)

[7 研究步骤 18](#_Toc63595091)

[7.1 筛选期 18](#_Toc63595092)

[7.3 研究治疗结束/退出研究 23](#_Toc63595093)

[8 有效性评价 24](#_Toc63595094)

[9 不良事件（AE）和严重不良事件（SAE）的收集和报告 24](#_Toc63595095)

[9.1 不良事件 24](#_Toc63595096)

[9.2 不良事件的定义 24](#_Toc63595097)

[10 研究管理 28](#_Toc63595098)

[10.1 伦理规范及知情同意 28](#_Toc63595099)

[10.2 试验药物管理 29](#_Toc63595100)

[10.3 方案的修订 29](#_Toc63595101)

[10.4 监查 29](#_Toc63595102)

[10.5 质量控制和保证 29](#_Toc63595103)

[10.6 方案违背 30](#_Toc63595104)

[10.7 资料的保存 30](#_Toc63595105)

[10.8 研究结果的发表 30](#_Toc63595106)

[11 数据分析与统计方法 30](#_Toc63595107)

[11.1 样本量计算 30](#_Toc63595108)

[11.2 统计分析计划 30](#_Toc63595109)

[11.2.1 统计分析数据集 30](#_Toc63595110)

[11.2.2 统计分析方法 30](#_Toc63595111)

[11.2.3 统计软件 31](#_Toc63595112)

[12 病例脱落 31](#_Toc63595113)

[13 研究成果的发表形式 31](#_Toc63595114)

[附件一 乳腺癌的临床分期标准（第八版AJCC乳腺癌TNM分期） 33](#_Toc63595115)

[附件二 身体状况评分标准（ECOG） 33](#_Toc63595116)

[附件三 肌酐清除率的计算 33](#_Toc63595117)

[附件四 不良反应评价标准（NCI CTC AE 5.0版本，部分内容） 34](#_Toc63595118)

[附件五 RANO-BM疗效评价标准[11] 36](#_Toc63595119)

[附件六 输注相关反应管理 36](#_Toc63595120)

[参考文献 37](#_Toc63595121)

# 方案摘要

| **研究题目** | 重组人源化抗HER2单抗-AS269偶联药物（ARX788）治疗HER2阳性乳腺癌伴脑转移患者的前瞻性、单臂、单中心II期临床研究 |
| --- | --- |
| **研究申办单位** | 复旦大学附属肿瘤医院 |
| **主要研究者** | 胡夕春、张剑 |
| **版本号/日期** | 1.0/2021年02月19日 |
| **研究药物** | 重组人源化抗HER2单抗-AS269偶联药物（ARX788） |
| **研究对象** | HER2阳性晚期乳腺癌患者 |
| **研究目的** | 探索HER2阳性晚期乳腺癌伴脑转移患者的治疗策略：ARX788在既往使用过EGFR-TKI的HER2阳性乳腺癌伴脑转移患者中的疗效和安全性； |
| **终点指标** | **主要终点指标**：  颅内临床获益率（CNS CBR），根据RANO-BM标准  **次要终点指标**：  有效性指标：无进展生存期（PFS）；总生存期（OS）；首次进展部位  安全性指标：不良事件（AE），根据 NCI-CTC AE 5.0 标准  **探索性研究：**  收集并保存肿瘤样本（原发灶和/或转移灶）白片≥10张，基线、2疗程和疾病进展时（无论颅外病灶进展和/或颅内病灶进展）的抗凝血和促凝血各8ml，送我院精准检测中心检测HER2表达水平、包括HER2基因突变在内的肿瘤基因突变、PD-L1、TILs和ctDNA，用以探索性地研究可能影响或预测ARX788的疗效因素。 |
| **研究设计** | 本研究为开放的前瞻性、单臂、单中心II期临床研究。  本研究采用Simon两阶段设计，受试者接受治疗直至疾病进展、毒性不可耐受、退出研究、研究者判断必须终止用药，并收集药物有效性和安全性数据。疾病进展定义为颅内病灶进展（根据RANO-BM标准判断）和/或颅外病灶进展（根据RECIST 1.1标准判断）。终止治疗后医师根据受试者情况给出后续治疗建议，具体参考下表。  后续抗肿瘤治疗推荐建议   \| 疾病进展部位（事件） \| \| \| \| --- \| --- \| --- \| \| 颅内 \| 颅外 \| 治疗策略 \| \| 是 \| 是 \| 由研究医师决定后续治疗 \| \| 是 \| 否 \| 继续试验方案治疗+脑局部治疗* \| \| 否 \| 是 \| 由研究医师决定后续治疗 \| \| 否 \| 否 \| 继续按试验方案治疗 \|   备注：研究方案规定，受试者接受试验药物方案治疗直至疾病进展、毒性不可耐受、退出研究或研究者判定必须终止用药。其中疾病进展定义为颅内病灶进展或颅外病灶进展或颅内和颅内病灶均进展。充分考虑研究药物疾病控制特点以及受试者获益最大化，在区别颅内、颅外病灶进展的情况下制定了该表内容，*：若不能进行脑局部治疗，则由研究医师决定后续治疗。  终止治疗的受试者即进入随访期：1）安全性随访至研究药物末次给药后28天；2）非疾病进展、非死亡原因终止用药的受试者需接受疗效随访直至疾病进展、开始接受其他抗肿瘤药物治疗或死亡，以先达到者为准；3）所有受试者接受生存随访直至死亡或OS数据收集结束（以先出现者为准）。  颅内CBR评价标准参考RANO-BM标准，颅外病灶的肿瘤影像学评价依据RECIST 1.1标准。  治疗方案：ARX788 1.5mg/kg，d1，q3w |
| **入组标准** | 受试者必须满足以下所有入选标准才可入组本试验：   - - - 1. 年龄≥18岁，且≤75岁，性别不限；       2. ECOG评分0-2分；       3. 病理检测确诊为HR任意/HER2阳性的乳腺癌患者；HER2阳性定义为：免疫组化检测HER2（3+）或者荧光原位杂交技术（FISH）检测结果为阳性；       4. 既往接受过曲妥珠单抗、紫杉类和EGFR-TKI类药物治疗；       5. MRI证实存在脑转移，且至少有一个最长径≥1.0 cm的颅内脑实质转移病灶；该转移灶可以是新诊断且未经治疗的脑转移灶，或在既往局部治疗后出现进展的脑转移灶，但在进展后尚未再次接受局部干预治疗；       6. 入组前允许使用甘露醇、贝伐珠单抗或激素治疗，但药物治疗剂量至少能够稳定一周不需要增量；       7. 器官的功能水平必须符合下列要求：   1） 血常规  • ANC≥1.5×10^9^/L；  • PLT≥75×10^9^/L；  • Hb≥90 g/L（允许输血或使用药物治疗保证血红蛋白含量）；  2） 凝血功能：INR≤1.5，APTT≤1.5×ULN；  3）血生化  • TBIL≤1.5×ULN；  • ALT和AST≤3×ULN（肝转移≤5.0×ULN）；  • Cr≤1.5×ULN或肌酐清除率≥50 mL/min （Cockcroft-Gault公式）；  4） 心脏彩超：LVEF≥50%；  5） 12导联心电图：Fridericia 法校正的 QT 间期（QTcF）女性＜470ms，男性＜450ms；   1. 首次给予研究药物前患者已从既往外科手术和既往癌症治疗相关的任何AE中恢复（≤1级），以下情况除外：a. 脱发；b. 色素沉着；c. 放疗引起的远期毒性，经研究者判断不能恢复；d. 铂类药物引起的；2级及以下神经毒性； 2. 自愿加入本研究，签署知情同意，有良好的依从性并愿意配合随访。 |
| **排除标准** | 凡有下列情况之一，不入选为受试者：   1. 经MRI或腰穿证实的软脑膜转移或囊性转移病灶； 2. 存在无法通过引流或其他方法控制的第三间隙积液（如大量胸水和腹水）； 3. 既往接受过T-DM1或其他HER2-ADC类药物治疗者； 4. 试验药物治疗前2周内接受过全脑放疗、化疗、手术，前一周内接受过曲妥珠单抗靶向治疗或内分泌治疗，前2周内接受过针对骨转移的姑息性放疗者； 5. 既往存在需要激素治疗的肺间质性疾病、药物所致肺间质性疾病、放射性肺炎病史，或目前任何证据提示的临床活动性肺间质性疾病者； 6. 现患角膜炎、角膜疾病、视网膜疾病或活动性眼部感染等需要干预的眼部疾病者； 7. 不愿或不能在试验期间停止佩戴角膜接触镜者； 8. 入组前2周内参加过其它药物临床试验； 9. 同时接受其他任何瘤肿的任何抗肿瘤治疗者，以控制脑水肿为目的的贝伐珠单抗以及治疗骨转移或预防骨质疏松为目的的双膦酸盐除外； 10. 既往5年内患有其他恶性肿瘤，不包括已治愈的宫颈原位癌、皮肤基底细胞癌或皮肤鳞状细胞癌； 11. 心功能不全受试者，包括但不限于充血性心力衰竭、透壁性心肌梗死受试者、需要药物治疗的心绞痛、临床上显著的心脏瓣膜病及高危心律失常或筛选期ECG检查中QTc异常且有临床意义（静息状态下，ECG检查校正后QTc＞450 msec[男]或QTc＞470 msec[女]）； 12. 未控制的高血压（静息状态下：收缩压＞160 mmHg或舒张压＞100 mmHg）； 13. 既往暴露于蒽环类药物的累积达到以下剂量：     - 多柔比星或脂质体多柔比星>500 mg/m^2^；     - 表柔比星>900 mg/m^2^；     - 米托蒽醌>120 mg/m^2^；     - 其他（即脂质体多柔比星或其他蒽环类药物>相当于500 mg/m^2^的阿霉素）；     - 如果使用多于一种蒽环类药物，那么累积剂量不得超过相当于500 mg/m^2^的阿霉素。 14. 已知对ARX788的任一活性成分或辅料过敏者，或有明确蛋白类药物过敏史、特异性变态反应病史（哮喘、风湿、湿疹性皮炎）或曾发生过其它严重的过敏反应且经研究者判断不适合接受ARX788治疗者； 15. 有免疫缺陷病史，包括 HIV 检测阳性，或患有其他获得性、先天性免疫缺陷疾病，或有器官移植史； 16. 乙肝表面抗原阳性且HBV DNA≥1000者，或丙型肝炎病毒抗体、梅毒螺旋体抗体、人类免疫缺陷病毒抗体检查结果中任意一种呈阳性者； 17. 既往有明确的神经或精神障碍史，包括癫痫或痴呆； 18. 妊娠期、哺乳期女性患者，有生育能力且基线妊娠试验阳性的女性患者或在整个试验期间不愿意采取有效避孕措施的患者； 19. 根据研究者的判断，有严重的危害患者安全、或影响患者完成研究的伴随疾病（包括但不限于药物无法控制的严重高血压、严重的糖尿病、活动性感染、甲状腺疾病等）； 20. 研究者认为患者不适合参加本研究的其他任何情况。 |
| **数据分析与**  **统计方法** | 根据本研究主要终点指标CNS CBR估计样本量，预估本研究入组为既往已经接受过抗HER2 TKI的HER2阳性难治性脑转移乳腺癌患者，因这部分患者的CNS CBR缺乏文献报道，预估为10%，假设ARX788方案治疗组可以将CNS CBR提高至30%。利用simon两阶段设计方法进行样本量计算，双侧检验，I类错误5%，把握度80%，第一阶段共入组10例，若≤1例受试者出现CR/PR/SD≥24周，则终止研究，并判定该治疗方案无效，否则按照计划完成29例受试者入组，若≤5例受试者出现CR/PR/SD≥24周，则判断为该治疗方案无效，否则认为该治疗方案有效。考虑失访率为10%，一共需要入组32例受试者。   - 有效性分析   主要终点为CNS CBR，其分析将基于FAS和PPS集，其中FAS为主要分析集。描述性分析无进展生存期（PFS）；总生存期（OS）；首次进展部位。   - 安全性分析   安全性分析将基于SS集。所有不良事件将按照NCI-CTC AE 5.0版进行分级。以描述性统计分析为主，根据组别对AE、SAE、≥3级的AE、≥3级的SAE、与药物相关的AE、与药物相关的SAE、发生率≥5%的AE、发生率≥5%的SAE、导致剂量调整的AE、导致终止治疗的AE等数据进行统计汇总。实验室检验结果、生命体征、心电图及超声心动图等数据将采用转换表对基线与基线后的情况进行分析。 |
| **研究进度** | 预计首例受试者入组时间：2021年05月  预计末例受试者入组时间：2022年05月  预计末例受试者出组时间：2022年11月  预计研究结束时间：2023年05月 |

临床试验流程图

| **研究日/检查或操作** | **筛选期** | **治疗期** | | | | **停药随访期** | **长期随访期^15^** |
| --- | --- | --- | --- | --- | --- | --- | --- |
|  |  | **第1周期（C1）** | | | **第2周期（C2）及以后^14^** |  |  |
|  | **D-28~D-1** | **D1** | **D7±1天** | **D14±3天** | **±3天** | **停药后28＋7天** | **±7天** |
| **签署知情同意书** | X |  |  |  |  |  |  |
| **人口统计学信息** | X |  |  |  |  |  |  |
| **病史收集^1^** | X |  |  |  |  |  |  |
| **治疗史收集^2^** | X |  |  |  |  |  |  |
| **入选/排除标准判断** | X |  |  |  |  |  |  |
| **眼科检查^3^** | X |  |  |  |  |  |  |
| **生命体征** | X | X^4^ | X | X | X | X |  |
| **体格检查** | X | X |  |  | X | X |  |
| **血常规** | X**^5^** |  | X | X | X | X |  |
| **血生化** | X**^5^** |  | X | X | X | X |  |
| **尿常规** | X**^5^** |  |  |  | X | X |  |
| **12导联心电图** | X^5^ | X^6^ | X | X | X | X |  |
| **ECOG评分** | X |  |  |  | X | X |  |
| **妊娠检查（仅限育龄女性）** | X |  |  |  | X | X |  |
| **凝血功能检查** | X |  |  |  | X | X |  |
| **超声心动图** | X |  |  |  | X | X |  |
| **血清病毒学检查^7^** |  |  |  |  |  |  |  |
| **乙肝五项检查±HBV DNA** | X^7^ |  |  |  | X | X |  |
| **HCV抗体** | X^7^ |  |  |  |  |  |  |
| **HIV抗体** | X^7^ |  |  |  |  |  |  |
| **梅毒螺旋体** | X^7^ |  |  |  |  |  |  |
| **影像学检查^8^** |  |  |  |  |  |  |  |
| **胸部、腹部、盆腔增强CT或MRI** | X |  |  |  | X |  | X |
| **头颅MRI** | X |  |  |  | X |  | X |
| **骨放射性核素扫描（ECT）** | X |  |  |  |  |  |  |
| **颅外病灶参考RECIST 1.1标准/颅内病灶参考RANO-BM标准^9^** | X |  |  |  | X |  | X |
| **体重^10^** |  | X |  |  | X |  |  |
| **试验用药品给药** |  | X | | | |  |  |
| **输注反应监测^11^** |  | X |  |  | X |  |  |
| **样本采集^12^** |  | X | | | |  |  |
| **电话访视^13^** |  |  |  |  |  | X | |
| **合并用药记录** |  | X | | | | |  |
| **不良事件记录** | X | | | | | |  |

1. 病史收集，可包括但不限于：

- 经组织病理学或细胞学诊断为乳腺癌的历史数据；
- 分子分型诊断数据（可包括激素受体状态等）；
- 签署知情同意书前5年内恶性肿瘤病史；
- 既往或现在是否存在需要激素治疗的肺间质性疾病、药物所致肺间质性疾病、放射性肺炎病史，或任何证据提示的临床活动性肺间质性疾病；
- 既往或现在是否存在心功能不全，包括但不限于充血性心力衰竭、透壁性心肌梗死受试者、需要药物治疗的心绞痛、临床上显著的心脏瓣膜病及高危心律失常；
- 既往或现在是否存在严重或具有显著临床意义的全身性疾病，如心脏、肺、代谢或肝肾疾病等。

1. 治疗史收集，至少包括：

- 之前接受抗肿瘤治疗的情况；
- 随机前4周内的用药情况，至少包括糖皮质激素使用情况等。

1. 眼的一般检查，包括眼附属器和眼前段检查；
2. 首次试验用药品给药当天的生命体征检查分别在试验用药品给药前30 min内、给药后2 h±30 min各进行1次；
3. 筛选期血常规、血生化、尿常规检查及12导联心电图需要在开始给药前一周内完成，否则需在给药前一周内重新检测；
4. 首次试验用药品给药当天的12导联心电图检查分别在试验用药品给药前（可接受给药开始前48 h内检测结果）、给药后2 h±30 min各进行1次；
5. 血清病毒学检查，指标包括HBsAg、HBsAb、HBeAg、HBcAb、HBeAb、HBV DNA定量（仅在乙肝表面抗原阳性受试者中进行），HCV抗体检测，HIV抗体检测，梅毒螺旋体检测；治疗期间仅在筛选期乙肝表面抗原阳性受试者中进行乙肝五项检测，且由研究者判断是否需要进行HBV DNA定量检查；
6. 肿瘤影像学评价，主要包括：
   - 胸部、腹部、盆腔增强CT或MRI：根据初始扫描选择靶病灶和非靶病灶，所有后续扫描应该使用同一方法；对于造影剂过敏的患者，若经研究者判断后，可进行平扫CT或MRI；
   - 头颅MRI检查：为必须检查项目；
   - 骨放射性核素扫描（ECT）：筛选期中ECT检查为必须检查项目，治疗期间及长期随访访视时由研究者根据受试者的具体情况（如出现骨痛或者碱性磷酸酶升高）判断后进行；
   - 研究者可根据个体受试者的体征及状态，决定是否进行其他影像学检查，如乳腺MRI、乳腺X摄片、乳腺超声或其他怀疑转移灶处的影像学检查等。
7. 由研究者根据RECIST1.1版进行颅外病灶的疗效评估，根据RANO-BM进行颅内病灶的疗效评价。首次颅内/颅外PR/CR，须在至少4周后进行确认。
8. 每次给药前根据给药当天的体重重新计算药物剂量。
9. ARX788输注前后均应密切关注受试者的不良事件发生情况，并及时进行处理。若出现输注相关的症状（如发烧或寒颤等），可降低输注速度或中断输注；
10. 将在本研究试验药物组受试者中进行血样本和组织样本的采集，时间点为基线、2疗程、颅内和/或颅外疾病进展时。
11. 停药后受试者应完成停药随访期，研究医生应每周进行一次电话访视，询问受试者是否发生不良事件、是否使用其他药物或治疗手段，电话访视时间窗为±3天。期间若受试者发生任何不良事件或使用任何合并用药，需及时向研究者报告并记录在患者日记卡中，必要时可来院进行必要的干预。
12. 治疗期间，第2周期及以后各项检查及操作的频率如下：
    - 体重测量、生命体征、体格检查、血常规、血生化、尿常规及12导联心电图检查应在C2及之后每个周期试验用药品给药前3天内进行，其中试验药物组受试者还将在每次试验药物给药完成后2 h±30 min进行1次生命体征及12导联心电图检查；
    - 超声心动图、凝血功能、ECOG评分、血清病毒学（乙肝五项±HBV DNA）、妊娠检查应在C3及之后每2个周期试验用药品给药前3天内进行；
    - 肿瘤影像学检查及疗效评价自随机后每6周±3天进行一次。
13. 停药随访期后受试者进入长期随访期，以获得足够数据用于支持长期获益终点指标的分析及评价。研究者将每3个月（按照30天/月计算）进行一次电话访视，获知受试者是否接受其他抗肿瘤治疗、生存信息等，直至受试者死亡、退出研究、失访、拒绝电话随访或试验结束（包含试验完成及试验提前终止）。电话访视的时间窗为±7天。长期随访期内仅收集与试验药物相关的严重不良事件。生存随访期内，如果您未出现疾病进展，且没有接受其他抗肿瘤治疗，您需按照原计划时间（自随机后每6周）来院进行肿瘤疗效评价，包括肿瘤影像学检查（如CT、磁共振成像等）和评估，同时，研究医生将询问您自上次访视以来是否有任何变化或受到伤害。

# 缩略语表

| **缩略语** | **中文** |
| --- | --- |
| ADL (Activities of Daily Living) | 日常生活能力 |
| AE (adverse event) | 不良事件 |
| AI (aromataseinhibitor) | 芳香化酶抑制剂 |
| AJCC (American Joint Committee on Cancer) | 美国癌症联合委员会 |
| AKP/ALP (Alkaline Phosphatase) | 碱性磷酸酶 |
| ALB (albumin) | 白蛋白 |
| ALT (alanine aminotransferase) | 丙氨酸氨基转移酶 |
| ANC (absolute neutrophil count) | 中性粒细胞计数 |
| ASCO (American Society of Clinical Oncology) | 美国临床肿瘤学会 |
| AST (aspartate aminotransferase) | 天门冬氨酸氨基转移酶 |
| AUC (area under concentration time curve) | 药-时曲线下面积 |
| BMI (Body Mass Index) | 身体质量指数 |
| BUN (blood urea nitrogen) | 尿素氮 |
| CA125 | 糖类抗原125 |
| CAP (College of American Pathologists) | 美国病理学家协会 |
| CBR (clinical benefit rate) | 临床获益率 |
| CEP17 | 第17号染色体计数探针 |
| CD-ROM (Compact Disc Read-Only Memory) | 只读光盘 |
| CHOL (cholestenone) | 胆固醇 |
| CI (Confidence Interval) | 置信区间 |
| Cmax (peak concentration) | 峰浓度 |
| CFDA (China Food and Drug Administration) | 国家食品药品监督管理总局 |
| CL/F | 表观口服清除率 |
| Cr (creatinine) | 肌酐 |
| CR (complete response) | 完全缓解 |
| CRC (Clinical research coordinator) | 临床协调员 |
| CT (Computed Tomography) | 电子计算机断层扫描 |
| CTC (Common Terminology Criteria) | 通用毒性标准 |
| CDK4/6 (cyclin-dependent kinase4/6) | 周期蛋白依赖性激酶4/6 |
| CYP (cytochrome) | 细胞色素 |
| DBIL (direct bilirubin) | 直接胆红素 |
| DFS (Disease-freesurvival) | 无病生存期 |
| DLT (dose limited toxicity) | 剂量限制毒性 |
| DNA (deoxyribonucleic acid) | 脱氧核糖核酸 |
| DPD (dihydropyrimidine Dehydrogenase) | 二氢嘧啶脱氢酶 |
| EC (Ethics Committee) | 伦理委员会 |
| ECG (electrocardiogram) | 心电图 |
| ECOG (Eastern Cooperative Oncology Group) | 美国东部肿瘤协作组 |
| EGF (Epidermal Growth Factor） | 表皮生长因子 |
| EGFR (epidermal growth factor receptor) | 表皮生长因子受体 |
| ER (estrogen receptor) | 雌激素受体 |
| FAS (Full analysis set) | 全分析集 |
| FDG | 2-氟-2-脱氧-D-葡萄糖 |
| FISH (fluorescence in situ hybridization) | 荧光原位杂交技术 |
| g (gram) | 克/重量单位 |
| GCP (good clinical practice) | 临床试验规范 |
| h (hour) | 小时/时间单位 |
| Hb (Hemoglobin) | 血红蛋白 |
| HBV (hepatitis B virus) | 乙型肝炎病毒 |
| HCG (Human Chorionic Gonadotropin) | 人绒毛膜促性腺激素 |
| HCV (Hepatitis C Virus) | 丙型肝炎病毒 |
| HIV (Human Immunodeficiency Virus) | 人类免疫缺陷病毒 |
| HER2 (human epidermal growth factor receptor-2) | 人表皮生长因子受体-2 |
| HR (Hormone receptor) | 激素受体 |
| HR+ (Hormone receptor positive) | 激素受体阳性 |
| HR- (Hormone receptor negative) | 激素受体阴性 |
| IB (Investigator’s Brochure) | 研究者手册 |
| IBIL (indirect bilirubin) | 间接胆红素 |
| ICF (Informed consent form) | 知情同意书 |
| IC50 (50% Inhibition Concentration) | 半数抑制浓度 |
| IHC (Immunohistochemistry) | 免疫组织化学 |
| INR (international normalized ratio) | 国际标准化比值 |
| ISH (in situ hybridization) | 原位杂交 |
| ITT (intend to treat) | 意图治疗 |
| IU (international unit) | 国际单位/生物效应重量单位 |
| IV (intravenous) | 静脉注射（滴注） |
| kg (kilogram) | 千克 |
| L (Liter) | 升 |
| LC (lymphocyte cont) | 淋巴细胞计数 |
| LDH (lactate dehydrogenase) | 乳酸脱氢酶 |
| LVEF (Left Ventricular Ejection Fractions) | 左心室射血分数 |
| m (meter) | 米 |
| mL (Milliliter) | 毫升 |
| mg (milligram) | 毫克 |
| mg/m^2^ (milligram /square meter) | 毫克/平方米 |
| min (minute) | 分钟 |
| mm (millimeter) | 毫米 |
| ms (millisecond) | 毫秒 |
| MRI (Magnetic Resonance Imaging) | 磁共振成像 |
| MTD (maximum torlerate dose) | 最大耐受剂量 |
| NCI (national cancer institute) | 美国国立癌症研究所 |
| NCCN (National Comprehensive Cancer Network) | 美国国立综合癌症网络 |
| NE (not evaluate) | 无法评估 |
| NMPA (National Medical Products Administration) | 国家药品监督管理局 |
| non-pCR | 未达病理完全缓解率 |
| ORR (Objective Response Rate) | 客观缓解率 |
| OS (overall survival) | 总生存期 |
| pCR | 病理完全缓解率 |
| PD (progressive disease) | 疾病进展 |
| PET (positron emission tomography) | 正电子发射计算机断层显像 |
| PFS (progression-free survival) | 无进展生存期 |
| PI (Principal Investigator) | 主要研究者 |
| PK (pharmacokinetics) | 药代动力学 |
| PLT (blood platelet) | 血小板 |
| PPS (Per Protocol Set) | 符合方案分析集 |
| PR (partial response) | 部分缓解 |
| PgR (progesterone receptor) | 孕激素受体 |
| PSA (prostate specific antigen) | 前列腺特异抗原 |
| PT (prothrombin time) | 凝血酶原时间 |
| QTc | 按心率校正的QT间期 |
| RBC (red blood cell) | 红细胞计数 |
| RECIST (response evaluation criteria in solid tumors) | 实体瘤疗效评价标准 |
| RNA (Ribonucleic Acid) | 核糖核酸 |
| RTK (Receptor Tyrosine Kinase) | 受体酪氨酸激酶 |
| SAE (serious adverse event) | 严重不良事件 |
| SBP (systolic blood pressure) | 收缩期血压 |
| SIE (serious infection event) | 严重感染 |
| SD (stable disease) | 病情稳定 |
| SAP (statistical analysis plan) | 统计分析计划 |
| SS (Safety Set) | 安全性分析集 |
| T1/2 | 半衰期 |
| TBIL (total bilirubin) | 总胆红素 |
| TG (triglyceride) | 甘油三酯 |
| Tmax (peak time) | 达峰时间 |
| TP (plasma total protein) | 总蛋白 |
| TTP (Time to Progression) | 疾病进展时间 |
| μmol (micromole) | 微摩尔 |
| UNL (upper normal limit) | 正常值上限 |
| VEGF (Vascular Endothelial Growth Factor) | 血管内皮生长因子 |
| WBC (white blood cell) | 白细胞计数 |
| γGT (γ-glutamyl transpeptidase) | γ-谷氨酰转肽酶 |

# 1 研究背景

脑转移瘤是中枢神经系统恶性肿瘤常见的一种类型，发病率高出中枢神经系统原发恶性肿瘤4倍。所有恶性肿瘤患者约有20-40%最终会发生脑转移，其中位居第二的原发肿瘤是乳腺癌，仅次于肺癌[1]。目前不同分子分型的乳腺癌脑转移的发生率不同，所有转移性乳腺癌（Metastatic breast cancer, MBC）脑转移的发生率在5-15%左右[2, 3]，其中三阴性乳腺癌（triple-negative breast cancer, TNBC）和HER-2阳性乳腺癌的发生率最高，分别为30-45%和30-55%。乳腺癌患者一旦发生脑转移，生存期短，预后差，全部类型乳腺癌脑转移的中位生存期（median overall survival，mOS）仅为14个月。因此探索有效的治疗方案以改善乳腺癌脑转移患者的生存必要且迫切。

目前，HER2靶向治疗药物的出现使HER2阳性乳腺癌从具有不良预后的侵袭性疾病变为高度可治疗的疾病，即使在患有转移性疾病的患者中也可以延长存活期。对HER2生物学的深入理解增强了靶向治疗的发展，汇总数据显示[4]，已有4种HER2靶向治疗药物获得FDA批准用于HER2阳性转移性乳腺癌的治疗，主要包括曲妥珠单抗、Pertuzumab、T-DM1、和拉帕替尼，其中曲妥珠单抗及拉帕替尼也获得了我国药监机构的批准用于转移性乳腺癌的治疗。同时，我国自主研发的EGFR和HER2酪氨酸激酶双重抑制剂马来酸吡咯替尼，已获得NMPA的生产批准。

ARX788是由抗HER2单克隆抗体和毒素小分子AS269组成的抗体耦联药物，抗HER2单克隆抗体可与人HER2特异性结合，AS269为高效微管抑制剂，可抑制细胞生长。ARX788与T-DM1作用机制类似。ARX788单药在中国HER2晚期乳腺癌患者中进行的安全性、耐受性即药代动力学I期实验结果显示，试验药物的安全性较好，且经疗效评估后在1.5mg/kg q3w剂量组中的ORR高达69.2%，媲美DS8201a。既往研究显示，与ARX788类似的T-DM1在HER2阳性脑转移乳腺癌患者中的ORR在20-44%[5-8]。在未接受过治疗的HER2阳性乳腺癌脑转移患者中，EGFR-TKI类药物拉帕替尼联合卡培他滨的有效率高达66%[9]，而在难治性的HER2阳性乳腺癌脑转移患者中，拉帕替尼联合卡培他滨的ORR降至21%[10]。但是既往已经使用过EGFR-TKI后出现疾病进展的HER2阳性脑转移乳腺癌患者的治疗缺乏循证医学证据，如何治疗这部分患者成为临床难题。因此探索ADC药物在既往已经使用过EGFR-TKI HER2阳性脑转移乳腺癌患者中的疗效具有重要的临床意义。

因此，本研究拟开展II期临床试验，探索ARX788在HER2阳性晚期乳腺癌脑转移的治疗策略的疗效和安全性。

# 2 研究目的及终点指标

| **研究目的** | **研究终点** |
| --- | --- |
| **主要目的** | **主要终点指标** |
| 探索HER2阳性乳腺癌伴脑转移患者的治疗策略：ARX788在既往使用过EGFR-TKI的HER2阳性乳腺癌脑转移患者中的疗效和安全性； | 经研究者评估的颅内客观缓解率（CNS CBR），根据RANO-BM标准 |
|  | **次要终点指标** |
|  | 安全性指标：不良事件（AE），根据 NCI-CTC AE 5.0 标准  有效性指标：无进展生存期（PFS）；总生存期（OS）；首次进展部位 |

# 3 研究设计

本研究采用单中心、前瞻性、多队列、Simon两阶段设计，受试者接受治疗直至疾病进展、毒性不可耐受、退出研究、研究者判断必须终止用药，并收集药物有效性和安全性数据。

本试验为ARX788在HER2阳性晚期或转移性乳腺癌脑转移受试者中的单臂、开放、II期临床试验。根据本研究主要终点指标CNS CBR估计样本量，预估本研究入组多为既往已经接受过抗HER2 TKI的HER2阳性难治性脑转移乳腺癌患者，因这部分患者的CNS CBR缺乏文献报道，预估为10%，假设ARX788方案治疗组可以将CNS CBR提高至30%。利用simon两阶段设计方法进行样本量计算，双侧检验，I类错误5%，把握度80%，第一阶段共入组10例，若≤1例受试者出现CR/PR/SD≥24周，则终止研究，并判定该治疗方案无效，否则按照计划完成29例受试者入组，若≤5例受试者出现CR/PR/SD≥24周，则判断为该治疗方案无效，否则认为该治疗方案有效。考虑失访率为10%，一共需要入组32例受试者。

试验药物组ARX788的给药剂量设为1.5 mg/kg，每3周给药1次（Q3W），所有受试者将接受长期给药，直到出现不可耐受毒性或疾病进展或死亡或自愿退出或本试验结束（指试验完成或试验提前终止）。若获得ARX788其他临床试验最新信息后，研究者及申办方共同认为其他剂量可能具有更好的疗效且安全性可控，本研究试验药物组的部分或全部受试者可能使用其他给药方案。

治疗期间，所有受试者每6周接受一次抗肿瘤疗效评估，由研究者分别根据RECIST1.1标准和RANO-BM标准进行疾病状况判断，直至受试者出现疾病进展或死亡或拒绝来院随访或试验结束（包含试验完成及试验提前终止），以先发生事件为准。受试者出现疾病进展或拒绝来院随访后，研究者将每3个月（每个月按30天计算）进行一次电话访视，获知受试者的生存信息、是否接受其他抗肿瘤治疗等。收集本研究受试者的肿瘤样本（原发灶和/或转移灶）白片≥10张，基线、2疗程和疾病进展时（无论颅外病灶进展和/或颅内病灶进展）的抗凝血和促凝血各8ml，送我院精准检测中心检测HER2表达水平、包括HER2基因突变在内的肿瘤基因突变、PD-L1、TILs和ctDNA，用以探索性地研究可能影响或预测ARX788的疗效因素。

终止治疗的受试者即进入随访期：1）安全性随访至研究药物末次给药后28天；2）非疾病进展、非死亡原因终止用药的受试者需接受疗效随访直至疾病进展、开始接受其他抗肿瘤药物治疗或死亡，以先达到者为准；3）所有受试者接受生存随访直至死亡或OS数据收集结束（以先出现者为准）。

# 4 受试者的选择和退出

## 4.1 入组标准

受试者必须满足以下所有入选标准才可入组本试验：

- - - 1. 年龄≥18 岁，且≤75 岁，性别不限；
      2. ECOG评分0-2分；
      3. 病理检测确诊为HR任意/HER2阳性的乳腺癌患者；HER2阳性定义为：免疫组化检测HER2（3+）或者荧光原位杂交技术（FISH）检测结果为阳性；
      4. 既往接受过曲妥珠单抗、紫杉类和EGFR-TKI类药物治疗；
      5. MRI证实存在脑转移，且至少有一个既往未经放射治疗的最长径≥1.0 cm的颅内脑实质转移病灶；
      6. 入组前允许使用甘露醇、贝伐单抗或激素治疗，但药物治疗剂量至少能够稳定一周不需要增量；
      7. 器官的功能水平必须符合下列要求：

1） 血常规

• ANC≥1.5×10^9^/L；

• PLT≥75×10^9^/L；

• Hb≥90 g/L（允许输血或使用药物治疗保证血红蛋白含量）；

2） 凝血功能：INR≤1.5，APTT≤1.5×ULN；

3）血生化

• TBIL≤1.5×ULN；

• ALT和AST≤3×ULN（肝转移≤5.0×ULN）；

• Cr≤1.5×ULN或肌酐清除率≥50 mL/min （Cockcroft-Gault公式）；

4） 心脏彩超：LVEF≥50%；

5） 12导联心电图：Fridericia 法校正的 QT 间期（QTcF）女性＜470ms，男性＜450ms；

- - - 1. 首次给予研究药物前患者已从既往外科手术和既往癌症治疗相关的任何AE中恢复（≤1级），以下情况除外：a. 脱发；b. 色素沉着；c. 放疗引起的远期毒性，经研究者判断不能恢复；d. 铂类药物引起的；2级及以下神经毒性；
      2. 自愿加入本研究，签署知情同意，有良好的依从性并愿意配合随访。

## 4.2 排除标准

凡有下列情况之一，不入选为受试者：

1. 经MRI或腰穿证实的软脑膜转移或囊性转移病灶；
2. 存在无法通过引流或其他方法控制的第三间隙积液（如大量胸水和腹水）；
3. 既往接受过T-DM1或其他HER2-ADC类药物治疗者；
4. 试验药物治疗前2周内接受过全脑放疗、化疗、手术，前一周内接受过曲妥珠单抗靶向治疗或内分泌治疗，前2周内接受过针对骨转移的姑息性放疗者；
5. 既往存在需要激素治疗的肺间质性疾病、药物所致肺间质性疾病、放射性肺炎病史，或任何证据提示的临床活动性肺间质性疾病者；
6. 现患角膜炎、角膜疾病、视网膜疾病或活动性眼部感染等需要干预的眼部疾病者；
7. 不愿或不能再试验期间停止佩戴角膜接触镜者。
8. 入组前2周内参加过其它药物临床试验；
9. 同时接受其他任何瘤肿的任何抗肿瘤治疗者，以控制脑水肿为目的的贝伐珠单抗以及治疗骨转移或预防骨质疏松为目的的双膦酸盐除外；
10. 既往5年内患有其他恶性肿瘤，不包括已治愈的宫颈原位癌、皮肤基底细胞癌或皮肤鳞状细胞癌；
11. 心功能不全受试者，包括但不限于充血性心力衰竭、透壁性心肌梗死受试者、需要药物治疗的心绞痛、临床上显著的心脏瓣膜病及高危心律失常或筛选期ECG检查中QTc异常且有临床意义（静息状态下，ECG检查校正后QTc＞450 msec[男]或QTc＞470 msec[女]）；
12. 未控制的高血压（静息状态下：收缩压＞160 mmHg或舒张压＞100 mmHg）；
13. 既往暴露于蒽环类药物的累积达到以下剂量：
    - 多柔比星或脂质体多柔比星>500 mg/m^2^；
    - 表柔比星>900 mg/m^2^；
    - 米托蒽醌>120 mg/m^2^；
    - 其他（即脂质体多柔比星或其他蒽环类药物>相当于500 mg/m^2^的阿霉素）；
    - 如果使用多于一种蒽环类药物，那么累积剂量不得超过相当于500 mg/m^2^的阿霉素。
14. 已知对ARX788的任一活性成分或辅料过敏者，或有明确蛋白类药物过敏史、特异性变态反应病史（哮喘、风湿、湿疹性皮炎）或曾发生过其它严重的过敏反应且经研究者判断不适合接受ARX788治疗者；
15. 有免疫缺陷病史，包括 HIV 检测阳性，或患有其他获得性、先天性免疫缺陷疾病，或有器官移植史；
16. 乙肝表面抗原阳性且HBV DNA≥1000者，或丙型肝炎病毒抗体、梅毒螺旋体抗体、人类免疫缺陷病毒抗体检查结果中任意一种呈阳性者；
17. 既往有明确的神经或精神障碍史，包括癫痫或痴呆；
18. 妊娠期、哺乳期女性患者，有生育能力且基线妊娠试验阳性的女性患者或在整个试验期间不愿意采取有效避孕措施的患者；
19. 根据研究者的判断，有严重的危害患者安全、或影响患者完成研究的伴随疾病（包括但不限于药物无法控制的严重高血压、严重的糖尿病、活动性感染、甲状腺疾病等）；
20. 研究者认为患者不适合参加本研究的其他任何情况。

## 4.3 退出标准

**4.3.1 受试者退出标准**

退出本临床研究：

- - - 1. 受试者任意时间主动退出研究；
      2. 入组后发现受试者违反入排标准。

终止研究治疗，但仍需按研究要求继续随访：

1. 医学影像学评估的疾病进展；

2. ARX788下调至0.88 mg/kg Q3W仍然无法耐受毒性者；

3. 出现任何临床不良事件、实验室检查异常或其他医疗状况，导致受试者继续用药可能不再获益；

4. 研究过程中，受试者发生妊娠事件；

5. 医学或伦理学原因影响研究继续进行；

6. 严重违背试验方案，研究者评估认为应该终止治疗者；

7. 研究者认为无法继续研究药物治疗的其他原因。

**4.3.2 退出受试者的处理**

务必尽力按照方案中完成研究治疗结束/退出研究访视，对于终止研究治疗的受试者，须按照随访期的规定，对患者进行安全性随访、疗效随访（如需要）和生存随访。

研究者可以根据患者实际情况，向其建议或者提供新的或替代的治疗方法。

## 4.4 终止标准

本研究在有充分理由的情况下可能会提前终止或暂停。如果本研究被提前终止或暂停，研究者必须立即告知受试者，主要研究者需书面通知研究中心的临床试验机构/伦理委员会，并阐述提前终止或暂停理由。

本研究终止标准包括但不限于以下：

1. 发现对受试者有非预期的、意义重大的或不可接受的风险；
2. 试验执行过程中发现方案有重大失误；
3. 研究药物/试验治疗无效，或继续试验是无意义的；
4. 由于诸如受试者入选严重滞后或频繁的方案偏差等原因，造成完成试验极其困难。

# 5 治疗药物

## 5.1 研究药物名称和来源

ARX788是一种人源化抗HER2单克隆抗体与AS269（小分子细胞毒素药物）共价结合形成的抗体药物偶联物，且偶联比例固定为1:2。试验药物剂型为注射用无菌粉末，主要药物信息如下：

- **名称：**注射用重组人源化抗HER2单抗-AS269偶联物
- **代号**：ARX788
- **规格**：50 mg/支
- **用法用量**：静脉输注给药，1.5 mg/kg，Q3W
- **保存条件**：2℃~8℃，避光保存
- **运输、保存条件**：2℃~8℃ 冷链运输
- **有效期**：暂定24个月
- **辅料成分**：组氨酸、组氨酸盐酸盐、海藻糖和聚山梨酯80
- **提供方**：浙江新码生物医药有限公司

## 5.2 ARX788的给药方式

### ARX788：给药剂量为1.5 mg/kg，首次给药输注时间为90±10 min，如果耐受性良好，且无输液反应，则根据研究者的判断，后续输液可缩短到60±10 min。每周期的第1天给药，按照Q3W长期治疗。每个周期重新测量体重，若较上次测量或基线时体重变化＞±10%（以差异大者为准），则需重新计算具体用药量，且重新计算药物剂量时需将新的给药剂量下的体重作为后续计算的基线。

## 5.3 ARX788的暂停和剂量下调

若受试者出现具有显著临床意义的不良事件（AE）、或发生不可耐受的毒性，可接受必要的干预及支持治疗。试验药物组的受试者将根据治疗后发生不良事件级别、恢复情况及与ARX788的相关性，由研究者参考方案中规定的相关标准判断受试者是否需要进行ARX788的剂量调整、延迟给药或受试者退出研究。ARX788组受试者基于毒性的剂量调整可参考下表：

**表3 ARX788组受试者基于毒性的剂量调整**

| **AE** | **等级** | **剂量调整原则** |
| --- | --- | --- |
| 转氨酶上升（AST/ALT） | 2级（＞3、≤5倍正常值上限[ULN]） | 不进行剂量调整 |
|  | 3级（＞5、≤20倍ULN） | 暂停ARX788使用，AST/ALT水平恢复到≤2级时，降低剂量继续给药。 |
|  | 4级（＞20倍ULN） | 永久停止ARX788治疗 |
| 胆红素升高 | 2级（＞1.5、≤3倍ULN） | 暂停ARX788使用，总胆红素水平恢复到≤1级时，以原给药剂量继续给药。 |
|  | 3级（＞3、≤10倍ULN） | 暂停ARX788使用，总胆红素水平恢复到≤1级时，降低剂量继续给药。 |
|  | 4级（＞10倍ULN） | 永久停止ARX788治疗 |
| 血小板减少 | 2级（50,000 至75,000/mm^3^） | 2周内未恢复到≤1级或在此期间加重至3级，则暂停ARX788使用，血小板水平恢复到≤1级（≥75,000/mm^3^）时，以原给药剂量继续给药。 |
|  | 3级（25,000 至50,000/mm^3^） | 暂停ARX788使用，血小板水平恢复到≤1级（≥75,000/mm^3^）时，以原给药剂量继续给药。 |
|  | 4级（＜25,000 /mm^3^） | 暂停ARX788使用，血小板水平恢复到≤1级（≥75,000/mm^3^）时，降低剂量继续给药。 |
| 左心室功能障碍 | 症状性充血性心力衰竭 | 永久停止ARX788使用 |
|  | LVEF < 40% | 暂停ARX788使用，3周内重新评估LVEF，重测后仍LVEF < 40%，则永久停止ARX788使用。 |
|  | LVEF为40％至≤45％，与基线相比降低≥10％ | 暂停ARX788使用，3周内重新评估LVEF，重测后LVEF没有恢复到较基线相比10％以内，则永久停止ARX788使用。 |
|  | LVEF为40％至≤45％，与基线相比下降<10％ | 继续原剂量ARX788使用，加强LVEF监测。 |
|  | LVEF > 45% | 继续原剂量ARX788使用 |
| 肺部毒性（间质性肺病或肺炎等） | 1~2级 | 由研究者判断后可暂停试验药物治疗，如果12周内事件缓解至≤1级，研究者判断后可重新开始ARX788治疗；否则永久停止ARX788治疗，受试者退出研究； |
|  | 3级或4级 | 永久停止ARX788治疗，受试者退出研究。 |
| 眼部毒性（角膜炎或干眼症等） | 2级 | 如果2级眼部不良事件持续超过2周，则研究者判断后后续可降低剂量继续给药。如果2级眼部不良事件在减量后仍未缓解则暂停ARX788给药，如果12周内不良事件水平恢复到≤1级，研究者判定后可按照降低后剂量继续给药。 |
|  | 3级 | 暂停ARX788使用，如果12周内不良事件水平恢复到≤1级，研究者判定后可降低剂量继续给药。如果在试验药物减量后发生≥2级眼部不良事件，则永久停止ARX788治疗，受试者退出研究。 |
|  | 4级 | 永久停止ARX788治疗，受试者退出研究。 |
| 其他不良事件 |  | 由研究者结合受试者临床表现进行判断后，确定是否进行ARX788剂量调整、延迟给药或退出研究。 |

治疗期间，若受试者出现因毒性导致的剂量降低，则后续可使用降低后剂量进行持续治疗，受试者均不允许毒性恢复后进行剂量恢复。试验药物组最多允许受试者进行3个剂量水平的调整，具体剂量水平调整参考如下：

**表 4 ARX788剂量调整水平**

| **剂量水平** | **剂量** |
| --- | --- |
| 0 | 1.5 mg/kg Q3W |
| -1 | 1.3 mg/kg Q3W |
| -2 | 1.1 mg/kg Q3W |
| -3 | 0.88 mg/kg Q3W |
| 更低水平剂量调整 | 永久停药 |

方案中未明确规定的情况，需研究者考虑到受试者的利益/风险比再做决定。方案中已明确规定的情况，必须按规定中断/延迟治疗。

## 5.4 给药延迟

任何周期开始治疗前均应满足以下所有条件，否则需要延迟给药：

1. 中性粒细胞绝对值（ANC）≥1.5×10^9^/L

2. 血小板计数≥75×10^9^/L

3. 血红蛋白≥80g/L

## 5.5 试验药物的管理

根据GCP和国家法规要求，由浙江新码生物医药有限公司负责进行试验用药品的包装盒粘贴标签等工作，所有受试者的试验用药品均使用统一的包装。

试验标签的内容可包括：方案编号、申办方名称、包装规格、批号药物储藏条件、有效期等，具体包装以实物为准。

# 6 合并用药

试验过程中研究者可根据受试者的不良事件状态及临床需求使用支持治疗或辅助治疗。支持治疗可以包括但不限于止吐剂、阿片类或非阿片类镇痛剂、粒细胞集落刺激因子和红细胞生长因子等。

为充分暴露试验药物的安全性特征，在整个试验过程中不允许针对试验药物可能发生的不良事件（如皮疹、脱发、眼部毒性等）进行预防性用药。允许针对试验药物可能产生的输注反应进行预防用药，如为了预防受试者出现流感样输注反应，经研究者判断后可在试验药物输注开始前30 min给予临床常规预防输注用药（如非那根和/或吲哚美辛）。

受试者自进入本试验筛选直至整个治疗期间，除试验用药品外，不能同时接受任何抗癌药物治疗（用于骨转移治疗的双磷酸盐等药物除外），包括放疗、化疗、生物治疗、激素治疗或其他任何试验用药物，被批准用于抗肿瘤的中药在本试验过程中也不能使用。

研究中，受试者发生的所有AE、合并用药及治疗的原因、剂量/操作方法等均需完整记录在病史中。

# 7 研究步骤

本试验分为筛选期、治疗期、停药随访期、长期随访期，所有受试者均应按照方案在规定的时间完成相关访视及临床检查或观察。

## 7.1 筛选期

**7.1.1 筛选期（D-28~D-1）**

受试者或其法定代理人在所有筛选程序前必须本着自愿的原则签署知情同意书。符合全部入选标准且不符合排除标准的受试者方可参加试验。

筛选期内，受试者需接受的主要操作或检查包括：

- 签署知情同意书；
- 判断入选、排除标准；
- 采集人口学信息；
- 病史收集，可包括但不限于：
  - - 经组织病理学或细胞学诊断为乳腺癌的历史数据；
    - 分子分型诊断数据（可包括激素受体状态等）；
    - 签署知情同意书前5年内恶性肿瘤病史；
    - 既往或现在是否存在需要激素治疗的肺间质性疾病、药物所致肺间质性疾病、放射性肺炎病史，或任何证据提示的临床活动性肺间质性疾病；
    - 既往或现在是否存在心功能不全，包括但不限于充血性心力衰竭、透壁性心肌梗死受试者、需要药物治疗的心绞痛、临床上显著的心脏瓣膜病及高危心律失常；
    - 既往或现在是否存在严重或具有显著临床意义的全身性疾病，如心脏、肺、代谢或肝肾疾病等。
- 治疗史收集，至少包括：
  - - 之前接受抗肿瘤治疗的情况；
    - 首次研究用药前4周内的伴随治疗的情况。
- 眼科检查，眼的一般检查，包括眼附属器和眼前段检查；
- 生命体征；
- 体格检查；
- ECOG评分；
- 妊娠检查（仅限育龄女性）；
- 血常规；
- 血生化；
- 凝血功能检查；
- 尿常规；
- 12导联心电图；
- 超声心动图检查；
- 血清病毒学检查，可包括HBsAg、HBsAb、HBeAg、HBcAb、HBeAb、HBV DNA定量（仅在乙肝表面抗原阳性受试者中进行），HCV抗体检测，HIV抗体检测，梅毒螺旋体检测；
- 肿瘤影像学评价，所有影像学检查应在首次给药前28天内完成，且尽量靠近给药开始时间，主要包括：
- 胸部、腹部、盆腔增强CT或MRI：根据初始扫描选择靶病灶和非靶病灶，所有后续扫描应该使用同一方法；对于造影剂过敏的患者，若经研究者判断后，可进行平扫CT或MRI；
- 头颅MRI检查；
- 骨放射性核素扫描（ECT）：骨扫描检查可参照最近1年内的结果；若无最近1年内的骨扫描结果，则筛选期中ECT检查为必须检查项目，治疗期间由研究者根据受试者的具体情况（如出现骨痛或者碱性磷酸酶升高）判断后进行；
- 研究者可根据个体受试者的体征及状态，决定是否进行其他影像学检查，如乳腺MRI、乳腺X摄片、乳腺超声或其他怀疑转移灶处的影像学检查等。
- 根据实体瘤疗效评估标准（RECIST）1.1版评估颅外病灶，根据RANO-BM评估标准评估颅内病灶；首次颅内/颅外PR/CR，须在至少4周后进行确认；
- 合并用药记录；
- 不良事件记录。

D-1需对入/排标准进行复核。

**7.1.2 治疗期-第1天（C1D1）**

从C1D1开始，受试者按照方案规定的给药剂量及给药方案进行长期连续给药。C1D1受试者需要接受的检测及操作如下：

- 给药前体重测量；
- 血液样本采集（用于探索性研究）；
- 首次试验用药品给药，其中ARX788的输注时间为90±10分钟；
- 输注反应观察：ARX788输注期间及输注后应密切观察受试者的不良事件发生情况，并及时进行处理。若出现输注相关的症状（如发烧或寒颤），可降低输注速度或中断输注，具体处理措施可参考研究中心临床常规；
- 生命体征，分别在试验用药品首次给药前30 min内及给药完成后2 h±30 min进行；
- 体格检查（首次试验用药品给药前）；
- 12导联心电图；
- 合并用药记录；
- 不良事件记录。

**7.1.3 治疗期-第7天（C1D7天）**

治疗期间，受试者按照规定频率接受安全性及有效性检查，C1D7天所应接受的检查或操作如下：

- 生命体征；
- 血常规；
- 血生化；
- 12导联心电图；
- 合并用药记录；
- 不良事件记录。

C1D7访视的时间窗为±1天，任何超过方案规定时间的情况均应被记录。

**7.1.4 治疗期-第14天（C1D14）**

治疗期间，受试者按照规定频率接受安全性及有效性检查，C1D14天所应接受的检查或操作如下：

- 生命体征；
- 血常规；
- 血生化；
- 12导联心电图；
- 合并用药记录；
- 不良事件记录。

C1D14访视的时间窗为±3天，任何超过方案规定时间的情况均应被记录。

**7.1.5 治疗期-（C2D1~停药）**

治疗期间，以下给药操作或相关检查自首次试验用药品给药后每个周期进行一次，每个周期治疗访视的时间窗为±3天，应在完成每个周期所需进行的安全性检查且经研究者判断符合给药条件后再继续试验用药品治疗，任何超过方案规定时间的情况均应被记录。

- 体重测量，计算ARX788（按照体重）的具体用药量。
- 生命体征；
- 体格检查；
- 血常规；
- 血生化；
- 尿常规；
- 12导联心电图；
- 合并用药记录；
- 不良事件记录。

每个周期第1天，受试者应接受试验用药品给药，试验药物组受试者还应该进行输液相关反应监测、输注后（给药完成后2 h±30 min进行）生命体征和12导联心电图检查。若首次给药输注时耐受性良好，且无输液反应，则根据研究者的判断，后续ARX788的输注时间可缩短到60±10 min。若出现输注相关的症状（如发烧或寒颤），可降低输注速度或中断输注，具体处理措施可参考附件六输注相关反应管理。

以下检查或操作自首次试验用药品给药后每2个周期进行一次，访视的时间窗为±3天，任何超过方案规定时间的情况均应被记录。

- 生命体征；
- 体格检查；
- 12导联心电图；
- 血常规；
- 血生化；
- 尿常规；
- 凝血功能检查；
- 妊娠检查（仅限育龄女性）；
- 超声心动图；
- ECOG评分；
- 血清病毒学检查（乙肝五项±HBV DNA），仅在筛选期乙肝表面抗原阳性受试者中进行乙肝五项检测，且由研究者判断是否需要进行HBV DNA定量检查；
- 合并用药记录；
- 不良事件记录。

治疗期间，受试者自随机后每6周进行一次肿瘤疗效评价，时间窗为±3天，任何超过方案规定时间的情况均应被记录。主要包括以下操作：

- 肿瘤影像学评价，主要包括以下检查：
- 胸部、腹部、盆腔增强CT或MRI：根据初始扫描选择靶病灶和非靶病灶，所有后续扫描应该使用同一方法；对于造影剂过敏的患者，若经研究者判断后，可进行平扫CT或MRI；
- 头颅MRI检查；
- 骨放射性核素扫描（ECT）：筛选期中ECT检查为必须检查项目，治疗期间由研究者根据受试者的具体情况（如出现骨痛或者碱性磷酸酶升高）判断后进行；
- 研究者可根据个体受试者的体征及状态，决定是否进行其他影像学检查，如乳腺MRI、乳腺X摄片、乳腺超声或其他怀疑转移灶处的影像学检查等。
- 根据实体瘤疗效评估标准（RECIST）1.1版进行颅外病灶抗肿瘤疗效评价，根据RANO-BM标准进行颅内病灶抗肿瘤疗效评价；首次颅内/颅外PR/CR，须在至少4周后进行确认；

研究者可根据受试者体征或疾病状况增加检查，包括方案规定（每6周进行1次）之外的影像学检查，所有检查均应该被记录。

**7.1.6 停药随访期（停药后28＋7天内）**

受试者将持续用药直到出现不可耐受毒性或疾病进展或死亡或自愿退出或本试验结束。研究者可根据研究者评估的疾病进展情况决定受试者是否继续进行试验治疗。永久停止治疗后受试者将进入停药随访期。

停药后受试者应完成停药随访期，研究医生应每周进行一次电话访视，询问受试者是否发生不良事件、是否使用其他药物或治疗手段，电话访视的时间窗为±3天。受试者应在末次治疗后28天至医院接受末次治疗访视。期间若受试者发生任何不良事件或使用任何合并用药，需及时向研究者报告，必要时可来院进行必要的干预。若因发生与试验用药品相关的不良事件而永久停药，需随访至转归。

应在末次治疗后28天＋7天内应来院进行末次治疗访视，并完成以下检查：

- 体格检查；
- 生命体征；
- 血常规；
- 血生化；
- 尿常规；
- 12导联心电图；
- 超声心动图检查；
- ECOG评分；
- 妊娠检查（仅限育龄女性）；
- 凝血功能检查；
- 血清病毒学检查（乙肝五项±HBV DNA），仅在筛选期乙肝五项检测，且由研究者判断是否需要进行HBV DNA定量检查；

**7.1.7 长期随访期**

停药随访期后受试者进入长期随访期，以获得足够数据用于支持长期获益终点指标的分析及评价。长期随访期内，除非受试者因为评估的疾病进展或死亡而停药，否则受试者需按照原计划时间（自随机后每6周±3天）来院进行一次影像学疗效评估直到评估的疾病进展或死亡或拒绝来院随访或试验结束（包含试验完成及试验提前终止），主要包括：

- 肿瘤影像学评价：
- 胸部、腹部、盆腔增强CT或MRI：根据初始扫描选择靶病灶和非靶病灶，所有后续扫描应该使用同一方法；对于造影剂过敏的患者，若经研究者判断后，可进行平扫CT或MRI；
- 头颅MRI检查；
- 骨放射性核素扫描（ECT）：由研究者根据受试者的具体情况（如出现骨痛或者碱性磷酸酶升高）判断后进行；
- 研究者可根据个体受试者的体征及状态，决定是否进行其他影像学检查，如乳腺X摄片、乳腺超声或其他怀疑转移灶处的影像学检查等。
- 根据实体瘤疗效评估标准（RECIST）1.1版进行颅外病灶抗肿瘤疗效评价，根据RANO-BM标准进行颅内病灶抗肿瘤疗效评价；首次颅内/颅外PR/CR，须在至少4周后进行确认；

确定受试者疾病进展或受试者拒绝来院进行相关检查时，研究者将每3个月（按照30天/月计算）进行一次电话访视，获知受试者是否接受其他抗肿瘤治疗、生存信息等，直至受试者死亡、退出研究、失访、拒绝电话随访或试验结束（包含试验完成及试验提前终止）。电话访视的时间窗为±7天。长期随访期内仅收集与试验药物相关的严重不良事件。

**7.1.8 计划外访视**

出于对受试者安全性的考虑，研究者可要求受试者进行额外的访视或检查。

## 7.3 研究治疗结束/退出研究

受试者持续用药直至完成规定疗效，或疾病进展、毒性不可耐受、退出研究，或研究者判断必须终止用药。疾病进展定义为颅内病灶进展（根据RANO-BM标准判断）和/或颅外病灶进展（根据RECIST 1.1标准判断）。终止治疗后医师根据受试者情况给出后续治疗建议，具体参考下表8。

**表8 后续抗肿瘤治疗推荐建议**

| 疾病进展部位（事件） | | |
| --- | --- | --- |
| 颅内 | 颅外 | 治疗策略 |
| 是 | 是 | 由研究医师决定后续治疗 |
| 是 | 否 | 继续试验方案治疗+脑局部治疗* |
| 否 | 是 | 由研究医师决定后续治疗 |
| 否 | 否 | 继续按试验方案治疗 |

备注：研究方案规定，受试者接受试验药物方案治疗直至疾病进展、毒性不可耐受、退出研究或研究者判定必须终止用药。其中疾病进展定义为颅内病灶进展或颅外病灶进展或颅内和颅内病灶均进展。充分考虑研究药物疾病控制特点以及受试者获益最大化，在区别颅内、颅外病灶进展的情况下制定了该表内容，*：若不能进行脑局部治疗，则由研究医师决定后续治疗。

# 8 有效性评价

主要研究终点是分别探索各队列颅内客观缓解率（CNS CBR），根据RANO-BM标准，详见附件六。

次要研究终点是无进展生存期（PFS）；总生存期（OS）；首次进展部位。

# 9 不良事件（AE）和严重不良事件（SAE）的收集和报告

## 9.1 不良事件

AE信息的收集从受试者签署知情同意书开始，直至安全性随访期结束（末次用药后28天）。

## 9.2 不良事件的定义

AE是指临床试验受试者接受一种药品后出现的不良医学事件，但并不一定与治疗有因果关系。AE可以是任何不利的非期望的症状、体征、实验室检查异常或疾病等，至少包括以下几种情况： 1）原有的（进入临床试验之前）医学状况/疾病的加重 （包括症状、体征、实验室异常的加重）；2）新发生的任何AE：新发生的任何的不良医学状况（包括症状、体征、新诊断的疾病）；3）异常的具有临床意义的实验室检查值或者结果。

研究人员应详细记录受试者所发生的任何AE，包括：AE名称及所有相关症状的描述、发生时间、严重程度、与试验药物的相关性、持续时间、对研究用药采取的措施及最终结果和转归。

**9.2.1 不良事件严重程度判断标准**

参照 NCI-CTC AE 5.0版关于药物不良事件的分级标准。

如果出现 NCI-CTC AE 5.0 版表中未列出的不良事件可参照下列标准：

1级：轻度；无临床症状或有轻微临床症状；仅临床或诊断发现；无需治疗。

2级：中度；需要最小的、局部的或非侵入性的治疗；与年龄相符的工具性日常生活活动（Activities of Daily Living，ADL）受限。工具性日常生活活动是指做饭、购物、打电话、理财等。

3级：重度或有重要医学意义，但不会即刻危及生命；导致住院或住院时间延长；导致残疾；自理性日常生活活动（Self care ADL）受限。自理性日常生活活动是指：洗澡、穿衣、脱衣、吃饭、如厕、服药等，非卧床不起。

4级：具有危及生命的后果；需要紧急治疗。

5级：与不良事件有关的死亡。

**9.2.2 不良事件与试验药物关系的判断标准**

AE的收集从签署知情同意书开始，直至安全性随访期结束，不管事件是否与试验药物有关系，不管受试者是否分配入试验药物组，不管是否使用药物，均需收集记录。治疗期间受试者主诉的任何不适反应或客观实验室检查指标有异常改变，应如实记录，同时注明AE严重程度、持续时间、处理措施及转归等，研究者应综合判定AE与试验药物关系，例如AE的发生是否与用药有合理的时间顺序，研究药物特性，研究药物的毒理药理作用，受试者是否使用其他合并药物，受试者的基础疾病，病史，家族史以及激发和再激发反应等等。并按“肯定有关、可能有关、可能无关、肯定无关、无法判定”五级分类法对不良事件和试验用药之间可能存在的关联做出评估。判定标准如表10：

**表10 不良事件与试验药物关系的判断标准**

| **分级** | **判断标准** |
| --- | --- |
| 肯定有关 | 事件出现符合用药后合理的时间顺序，事件符合所疑药物已知的反应类型；停药后改善，重复给药再次出现该事件。 |
| 可能有关 | 事件出现符合用药后合理的时间顺序，事件不符合所疑药物已知的反应类型；病人的临床状态或其他治疗方式也有可能产生该事件。 |
| 可能无关 | 事件出现不符合用药后合理的时间顺序，事件不符合所疑药物已知的反应类型，病人的临床状态或其他治疗方式有可能产生该事件。 |
| 肯定无关 | 事件出现不符合用药后合理的时间顺序，事件不符合所疑药物已知的反应类型，病人的临床状态或其他治疗方式有可能产生该事件，疾病改善或停止其他治疗方式后事件消除，重复使用其他治疗方法事件出现。 |
| 无法评定 | 事件出现与用药后的时间顺序无明确关系，与该药品的已知的反应类型相似，同时使用的其他药物也可能引起相应的事件。 |

**9.2.3 严重不良事件**

严重不良事件（Serious adverse event，SAE）是指临床试验过程中发生需要住院治疗或延长住院时间、伤残、影响工作能力、危及生命或死亡、导致先天畸形等医学事件。符合下面一条或一条以上标准的医学事件都是 SAE：

• 导致死亡的事件；

• 危及生命的事件（定义为受试者在事件发生时有立即死亡的危险）；

• 导致住院治疗或延长住院时间的事件；

• 永久或严重致残/功能不全/影响工作能力的事件；

• 先天异常或出生缺陷；

• 其他重要医学事件：（定义为事件危害到受试者，或需要进行干预来预防上述任一情况的发生）。

**9.2.4 住院治疗**

临床研究中导致住院治疗（即使小于24小时）或住院时间延长的不良事件应视为SAE。

以下住院情况不构成 SAE：

• 康复机构

• 疗养院

• 常规急诊室收治

• 当日手术（如门诊/当日/非卧床的手术）

• 与不良事件恶化无关的住院治疗或住院时间延长本身不是严重不良事件。例如：

• 因原有疾病入院，并没有新的不良事件的发生，也没有原有疾病的加重（如：为了检查试

验前至今持续存在的实验室检查异常）；

• 管理原因的住院（如：每年例行的体检）；

• 临床试验期间试验方案规定的住院（如：按试验方案的要求进行操作）；

• 与不良事件恶化无关的择期住院（如：择期手术）；

• 已预定的治疗或外科手术应在整个试验方案和/或受试者个人的基线资料中予以记录；

• 仅因为血液品使用而入院。

诊断性或治疗性的侵入性（如手术）、非侵入性操作不应作为不良事件报告。但导致此项操作的疾病状况符合不良事件的定义时，应予以报告，如不良事件报告期间发病的急性阑尾炎应报告为不良事件，而因此进行的阑尾切除术应记录为该不良事件的治疗方法。

**9.2.5疾病进展和死亡**

疾病进展定义为研究的适应症引起的受试者情况恶化。包括影像学进展和临床症状、体征的进展。原发肿瘤的新转移灶，或原有转移灶的进展均认为是疾病进展。因疾病进展的症状和体征而引起的危及生命，需要住院治疗或延长住院时间，或导致永久性或严重残疾/功能不全/影响工作能力，先天异常或出生缺陷的事件，不作为SAE进行报告。如果对于SAE是否由于疾病进展所导致存在任何不确定性，则应报告为SAE。

在本试验的研究人群中，“疾病进展”是预期发生的情况，不应作为AE术语报告。当发生疾病进展时，用于确认疾病进展的事件应报告为AE。例如，受试者发生癫痫，被确定与脑转移有关，AE术语应记录为“癫痫”，而非“疾病进展”或“脑转移”。

若在试验期间受试者死亡，无论是否已接受新的抗肿瘤治疗，均必须作为SAE报告（参考表13 AE/SAE收集和随访期限原则）。经研究者评估可能由于疾病进展的症状和体征而引起的死亡应记录，且应作为SAE进行报告。“死亡”一词不应作为AE或SAE术语，而应作为事件的结果，引起或导致死亡的事件应记录为AE或SAE。如果死亡原因不明，且在报告时无法确定，则AE或SAE术语记录为“不明原因死亡”。

**9.2.6进行其他抗肿瘤治疗**

SAE的记录从受试者签署知情同意书开始直至安全性随访期结束（最后一次使用研究药物后28天）。若受试者在安全性随访期结束前开始进行其他抗肿瘤治疗，对于非死亡的SAE，除非怀疑与研究药物相关，其报告期限截止到开始新的抗肿瘤治疗。若死亡发生在安全性随访期内，无论受试者是否接受其他治疗，均必须作为SAE报告。

**9.2.7 SAE的报告制度**

SAE的收集期限应自受试者签署知情同意书始，直至安全性随访期结束。若发生SAE，无论是首次报告还是随访报告，研究者都必须立即填写《严重不良事件报告表》，签名及注明日期，在研究者获知24小时内立即报告伦理委员会和新码生物安全部门，并根据法规要求及时报告相关单位。新码生物安全部门接收SAE的报告邮箱地址为：xionggaozhun@zmc-china.com。

安全性随访期后发生的SAE，应对其中怀疑与研究药物有关者加以收集。SAE应详细记录症状、严重程度、与试验药物的相关性、发生时间、处理时间、采取措施、随访时间和方式以及转归情况。如果研究者认为某SAE与试验药无关，而与研究条件（例如终止原治疗，或试验过程中的合并症）潜在相关，则这种关系应在SAE报告表的叙述部分详细说明。如果某种正在发生的SAE的强度或其与受试药物的关系发生改变，应立即提交随访报告。如果研究者认为之前上报的SAE出现信息误报，可在随访报告中进行更正、撤销或降级说明，并按照SAE报告程序上报。

**9.2.8 特殊关注的不良事件**

特殊关注的不良事件包括与ARX788相关的以下肝毒性、血液学毒性、肺部毒性、眼部毒性、输注相关反应事件，具体包括：

- 肝毒性：3级及以上转氨酶（AST/ALT）升高、2级及以上胆红素升高；
- 血液学毒性：2级及以上血小板减少；
- 肺部毒性：包括但不限于非感染性肺炎或间质性肺炎等；
- 眼部毒性：包括但不限于干眼症或视物模糊等；
- 输注相关反应：包括但不限于发热、寒颤、僵直、发汗或头痛等。

**9.2.9** **妊娠**

临床研究期间女性受试者妊娠，则受试者出组，临床研究期间男性受试者的伴侣妊娠，则受试者继续临床研究。研究者应在获知妊娠事件后的24小时内填写《妊娠报告/随访表》并报告给新码生物安全部门，及时报告伦理。

研究者要对妊娠结果进行随访，随访至母亲分娩后1个月，并将结果报告给申办方。

如果妊娠结果为死产，自发性流产，胎儿畸形，则被视为SAE，需要按照SAE的时限要求报告。

如果受试者在妊娠期间同时发生SAE，则还要填写《严重不良事件报告表》，并须遵守SAE报告程序进行报告。

**9.2.10 AE/SAE的随访**

所有的AE/SAE均应随访至安全性随访期结束或消失、缓解至基线水平或≤ 1级、达到稳定状态，或得到合理解释（如失访、死亡）。

研究者应于每次访视时询问上次访视之后发生的AE/SAE情况，并依据申办方的质疑要求及时提供随访信息。在研究结束阶段，受试者末次用药后出现的AE/SAE收集与随访期限原则，可参考下表12：

**表12 AE/SAE收集和随访期限原则**

| **分类** | **收集/记录要求** | **随访要求** |
| --- | --- | --- |
| 无药物相关性的AE | 截止到安全性随访期结束或开始新的抗肿瘤治疗（以先达到者为准） | 截止到安全性随访期结束 |
| 有药物相关性的AE | 截止到安全性随访期结束 | 随访至消失、缓解，或至基线水平，或≤ 1 级、或达到稳定状态，或得到合理解释（如失访、死亡） |
| 无药物相关性的SAE | 截止到安全性随访期结束或开始新的抗肿瘤治疗（以先达到者为准） | 截止到安全性随访期结束 |
| 有药物相关性的SAE | 无限期 | 随访至消失、缓解，或至基线水平，或≤ 1 级、或达到稳定状态，或得到合理解释（如失访、死亡） |

AE：不良事件； SAE：严重不良事件

# 10 研究管理

## 10.1 伦理规范及知情同意

**10.1.1 伦理规范**

本临床试验必须遵循赫尔辛基宣言（2008 年版）、NMPA（原CFDA） 颁布的《药物临床试验质量管理规范》（GCP）以及相关的法规。在试验开始之前，必须获得负责单位伦理委员会批准，方可开始本项研究。在临床研究期间，本试验方案做一定的修改均应向伦理委员会报告并备案。研究者有责任依据伦理委员会相关要求定期递交试验期中报告，试验结束后应通知伦理委员会试验已经结束。

**10.1.2 知情同意**

受试者在接受本试验给药前必须对参加本试验知情同意，以保障受试者的合法权益。研究者有责任向受试者或其指定代表人完整、全面地介绍本研究的目的、药物的作用、可能出现的毒副反应和可能的风险，应让受试者知道他们的权利，所要承担的风险和受益。谈话是十分重要的知情同意过程。如受试者和其合法代表无识字能力，知情同意过程应有见证人参加，由受试者或其合法代表口头同意后，在知情同意书上签名，见证人的签名应与受试者的签名在同一天。知情同意书应注明版本编号和版本日期。

## 10.2 试验药物管理

本试验临床用药的管理、发放和回收由专人负责，研究者必须保证所有试验用药物仅用于参加该临床试验的受试者，其剂量与用法应遵照试验方案，剩余的药品退回申办者，不得把临床用药转交任何非临床试验参加者。

监查员负责对临床试验用药的供给、使用、储藏及剩余药品的处理过程进行监查。

## 10.3 方案的修订

除主要研究者外，任何人不可对方案进行修改。方案任何必须的改变，都需以方案修订形式进行，并需在获得主要研究者签字同意后提交伦理委员会审批或备案，同时在方案中说明历次修改的详细情况。

## 10.4 监查

研究者任命具备适当的医学、药学或相关专业学历，并经过必要的训练，熟悉GCP和有关法规的人员作为本临床试验的监查员，以监查和报告试验的进行情况和核实数据，保证临床试验中受试者的权益受到保障，试验记录与报告的数据准确、完整无误，保证 试验遵循已批准的方案、GCP 和有关法规。

通过对临床试验中不良事件和严重不良事件进行监查，以保证所有的不良事件均得到准确、可靠、迅速的记录和报告。

## 10.5 质量控制和保证

• 临床研究单位必须是 NMPA 确定的具有临床研究条件的药品临床研究基地；

• 研究人员必须是经过临床试验培训的医师，并在高级专业人员的指导下进行工作；

• 试验前检查临床病房必须符合规范化要求，保证抢救设备齐全；

• 由专业的护理人员给受试者用药，详细了解药物的服用情况，保证受试者的依从性；

• 各研究中心必须严格按研究方案进行；

• 监查员应遵循标准操作规程，监督临床试验的进行，确认所有数据的记录与报告正确完整，并与原始资料一致，保证试验按照临床研究方案执行；

• 一旦发生SAE，监查员须及时通报各试验单位，必要时需暂时停止该研究的进行；

• 参与试验的各中心应接受药品监督管理部门的稽查，尤为重要的是研究者及其相关人员应为监查和稽查提供方便和时间。

## 10.6 方案违背

研究方案中规定的所有要求，必须严格执行。任何有意或无意偏离或违反试验方案和GCP 原则的行为，均可归类为偏离方案或违反方案。监查员在监查过程中，如果发现偏离方案时应由研究者或监查员填写违反方案记录，详细记录发现的时间、事件发生的时间及过程、原因及相应的处理措施，由研究者签字，并通报伦理委员会。

## 10.7 资料的保存

为保证国家药品监督管理局和主要研究者对临床研究的评价与监督，研究者应同意保存所有研究资料，包括对受试者住院的原始记录、知情同意书、病例报告表、药品分发的详细记录等，研究资料应由研究机构保存至临床试验结束后 5 年。本临床研究的所有资料，所有权共同属于主要研究者及其所在机构复旦大学附属肿瘤医院和项目支持方浙江新码生物医药有限公司，除国家药品监督管理局要求外，未经所有人共同书面同意，任何人不得以任何形式提供给第三者。

## 10.8 研究结果的发表

所有与试验相关的文章和报道均需经过主要（研究者）研究单位同意后方可发表公开。

# 11 数据分析与统计方法

## 11.1 样本量计算

根据本研究主要终点指标CNS CBR估计样本量，预估本研究入组多为既往已经接受过抗HER2 TKI的HER2阳性难治性脑转移乳腺癌患者，因这部分患者的CNS CBR缺乏文献报道，预估为10%，假设ARX788方案治疗组可以将CNS CBR提高至30%。利用simon两阶段设计方法进行样本量计算，双侧检验，I类错误5%，把握度80%，第一阶段共入组10例，若≤1例受试者出现CR/PR/SD≥24周，则终止研究，并判定该治疗方案无效，否则按照计划完成29例受试者入组，若≤5例受试者出现CR/PR/SD≥24周，则判断为该治疗方案无效，否则认为该治疗方案有效。考虑失访率为10%，一共需要入组32例受试者。

## 11.2 统计分析计划

## 11.2.1 统计分析数据集

本试验药物的疗效分析针对全分析集（Full Analysis Set，FAS）和符合方案集（Per Protocol Set，PPS）进行。

本试验药物的安全性分析针对安全性分析集（Safety Set，SS）进行。

## 11.2.2 统计分析方法

**11.2.2.1 基本方法**

本研究中，如果不作特别说明，数据将按照以下一般原则采用描述性统计量进行汇总。

计量数据采用均数、标准差、中位数、最大值、最小值进行汇总；计数数据采用频数、百分比进行汇总，必要时给出百分比的95%置信区间；时间-事件数据采用Kaplan-Meier估计生存率并绘制生存曲线。

**11.2.2.2 有效性终点分析**

主要终点为CNS CBR，其分析将基于FAS和PPS集，其中FAS为主要分析集。描述性分析无进展生存期（PFS）；总生存期（OS）；首次进展部位。

**11.2.2.3 安全性分析**

所有不良事件（AEs）将按照NCI CTC AE 5.0版本分级系统进行分级。治疗中出现的不良事件（TEAE）定义为开始使用研究药物后新出现或较基线（研究治疗前）加重的任何不良事件。

安全性分析将以描述性统计汇总为主。根据组别对AE、SAE、≥3级的AE、≥3级的SAE、与药物相关的AE、与药物相关的SAE、发生率≥5%的AE、发生率≥5%的SAE、导致剂量调整的AE、导致终止治疗的AE等数据进行统计汇总。

分组描述实验室检验结果试验前正常但治疗后异常的情况以及发生异常改变时与试验药物的关系。

根据组别和访视对生命体征与基线的差值进行总结。

根据组别对ECOG-PS的基线和基线后最高评分进行总结。

根据组别对心电图、超声心动图的基线和基线后最差临床异常分级进行汇总。

安全性分析包括但不限于以上分析。

## 11.2.3 统计软件

采用 SAS9.4及以上版本分析。

# 12 病例脱落

所有填写了知情同意书并筛选合格进入试验的受试者，均有权随时退出临床试验。无论何时何因退出，只要没有完成临床试验1个疗程且无法进行安全性和有效性评价的受试者，均为脱落病例（入组后因疾病进展且有明确医学证据的不视为脱落）。当受试者脱落后，研究者必须填写脱落原因，完成所能完成的评估项目，并填写末次访视记录。对因不良反应而脱落，经随访最后判断与试验药物有关者，应记录并通知研究者。只进行了筛选而未取得药物编号而退出研究的受试者不作为脱落病例。如果受试者完成了一个完整疗程并有详细记录，则在作安全性评价时应统计分析。退出研究的受试者不能再次进入研究，其编号也不能再次使用。

# 附件一 乳腺癌的临床分期标准（第八版AJCC乳腺癌TNM分期）

| 0 期 | TisN0M0 |
| --- | --- |
| IA 期 | T1N0M0 |
| IB 期 | T0N1miM0 |
|  | T1N1miM0 |
| IIA 期 | T0N1M0 |
|  | T1N1M0 |
|  | T2N0M0 |
| IIB 期 | T2N1M0 |
|  | T3N0M0 |
| IIIA 期 | T0N2M0 |
|  | T1N2M0 |
|  | T2N2M0 |
|  | T3N1M0，T3N2M0 |
| IIIB 期 | T4N0M0，T4N1M0，T4N2M0 |
| IIIC 期 | 任何T，N3M0 |
| IV 期 | 任何T，任何N，M1 |

#

# 附件二 身体状况评分标准（ECOG）

（东部肿瘤协作组）

| 活动评分 | 描述 |
| --- | --- |
| 0 | 无症状，完全主动活动，及能够进行无限制的活动。 |
| 1 | 有症状，完全能行走，但重体力活动受限，能从事轻的或以坐为主的工作，如轻微家务、办公室工作。 |
| 2 | 有症状，能行走，生活可自理，但不能进行任何的体力活动，约有50%以上的时间清醒（白天卧床时间<50%）。 |
| 3 | 有症状，有限的生活自理能力，清醒时间卧床或坐椅>50%，但尚未卧床不起。 |
| 4 | 完全失去功能，生活完全不能自理，卧床不起。 |
| 5 | 死亡。 |

#

# 附件三 肌酐清除率的计算

内生肌酐清除率（Ccr）

应用 Cockcroft-Gault 公式公式计算：

Ccr(ml/min)(男性)=（140-年龄）×体重（kg）/［0.818×血清肌酐（µmol／L）］

Ccr(ml/min)(女性)= Ccr (男性) ×0.85

# 附件四 不良反应评价标准（NCI CTC AE 5.0版本，部分内容）


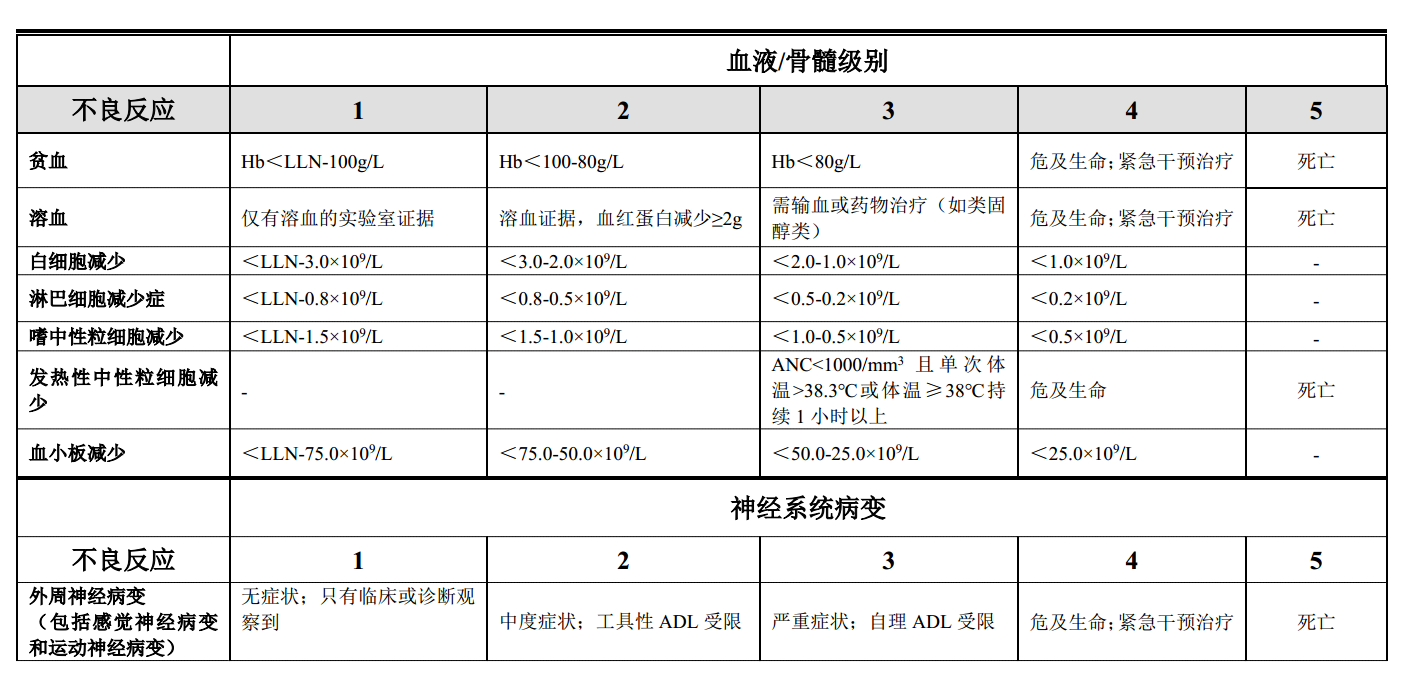


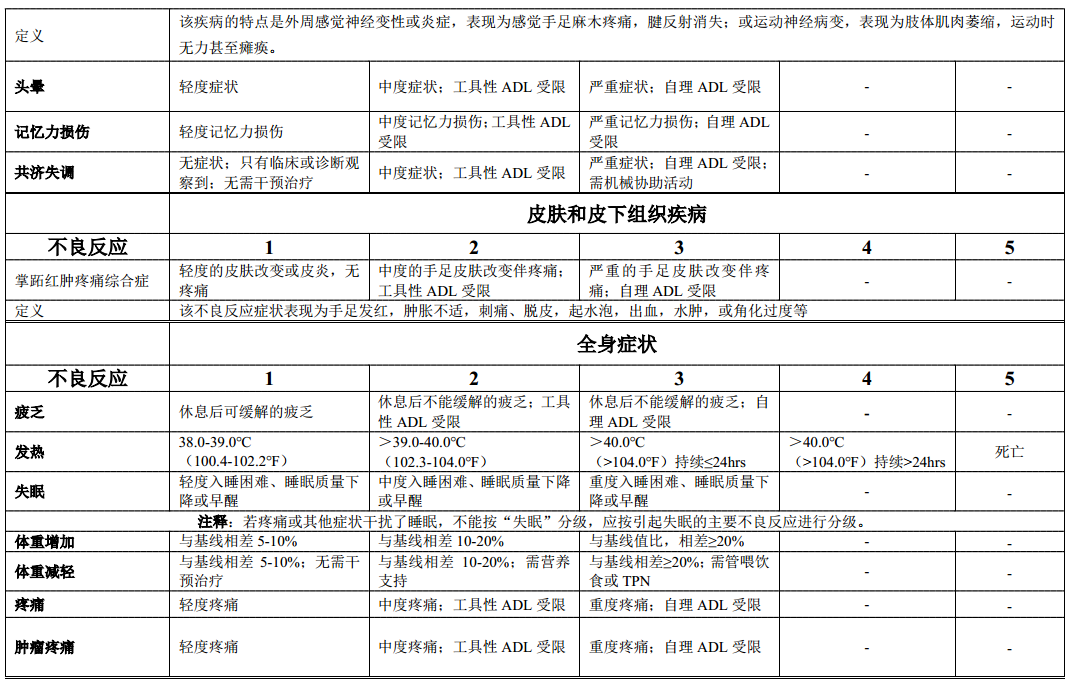


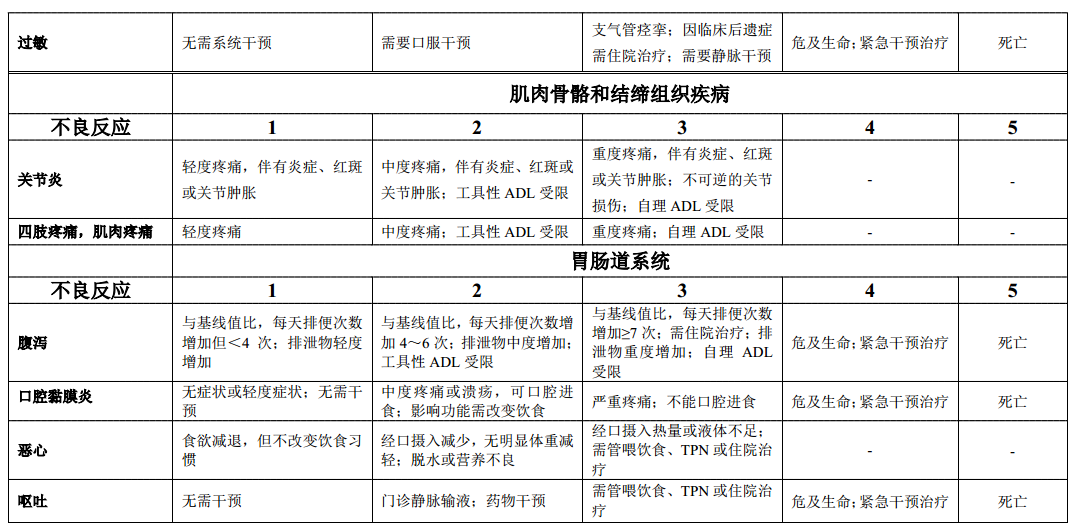


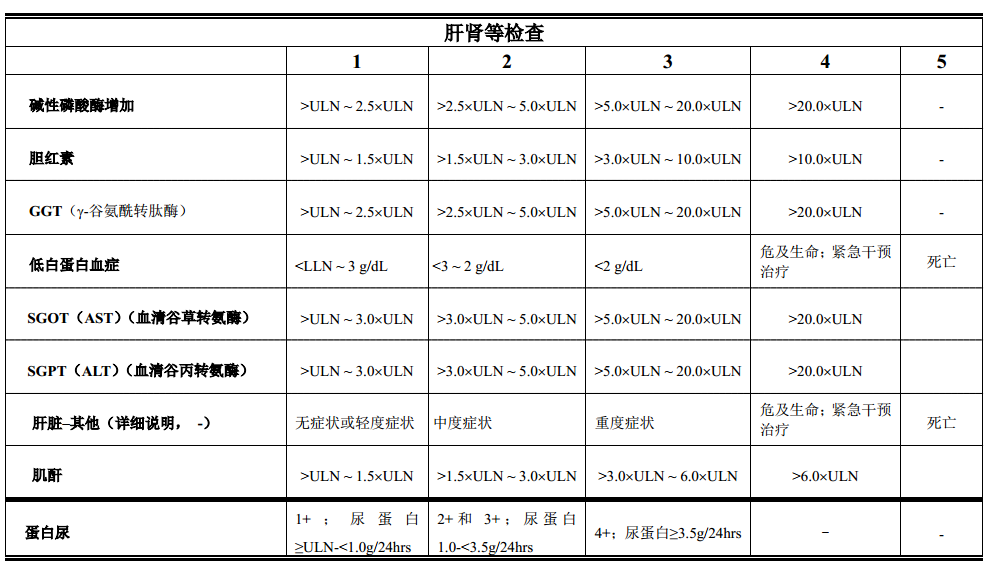


# 附件五 RANO-BM疗效评价标准[11]

**Table 1 Summary of the response criteria for CNS metastases proposed by RANO-BM**


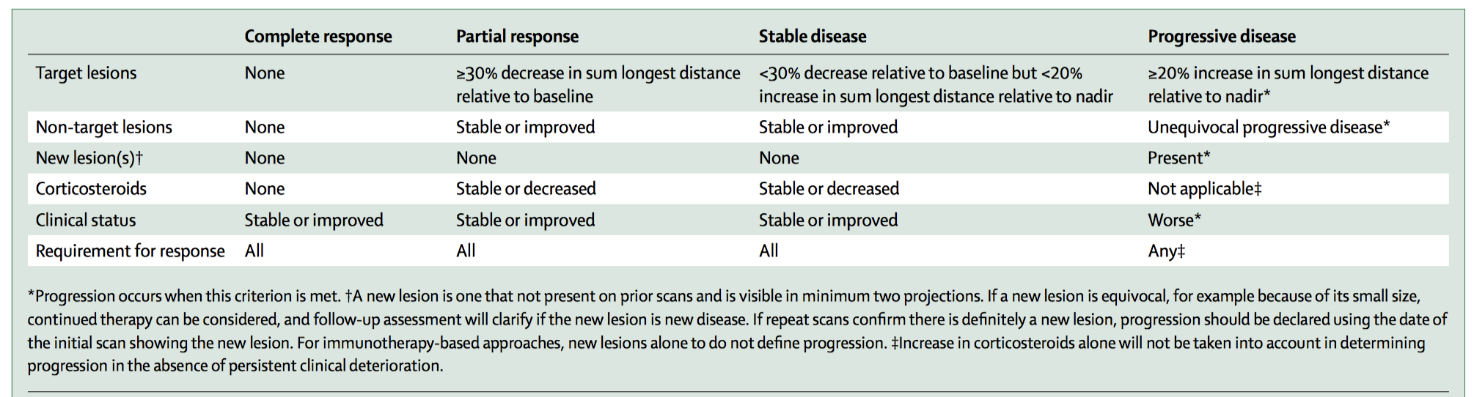


**Table 2 CNS and non-CNS response assessment**


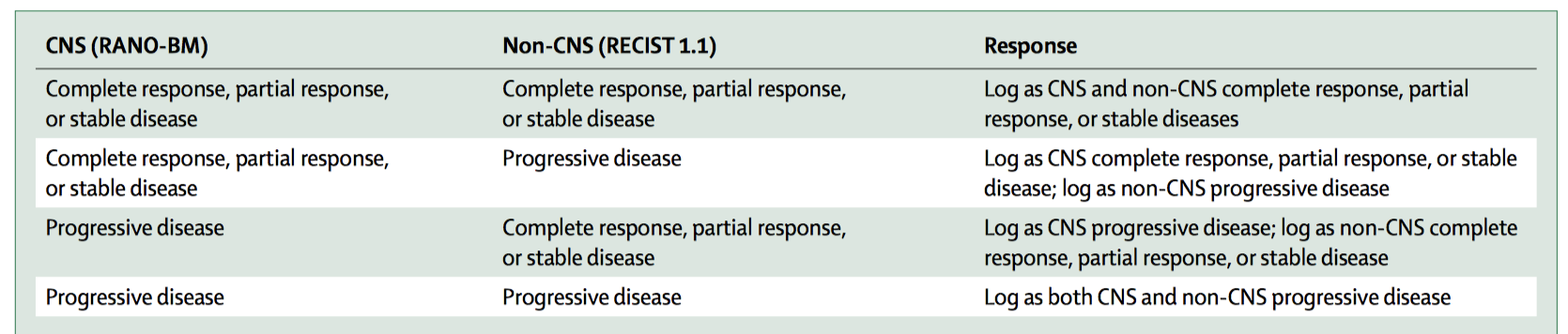


**Table 3 Bi-compartmental progression-free survival**


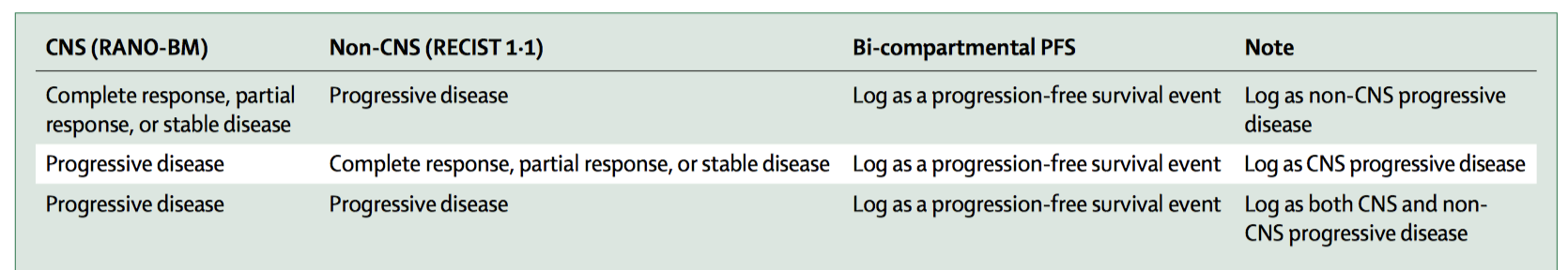


# 附件六 输注相关反应管理

输液相关反应的症状包括发热、寒战、僵直、发汗和头痛等。为了预防受试者出现流感样输注反应，经研究者判断后可在试验药物输注开始前30 min给予临床常规预防输注用药（如非那根和/或吲哚美辛）。对试验药物引起的输注反应症状的处理可参考如下方案：

- 1级-轻度：
- 轻度暂时性反应；
- 不需要中断输液；
- 不需要介入治疗。

此时应将研究药物的输液速度降低至当前输液速度约50%，并密切监测任何恶化情况。研究药物的总输液时间尽量不超过180 min，若180 min未完成输注且此时药物配置完成后未超过6小时，可将继续完成药液输注，否则应将剩余药液废弃，并记录实际用药量。

- 2级-中度：
- 需要中断治疗或输液，但对症治疗后立刻起效（如抗组胺药物、非甾体抗炎药物、麻醉药、IV液体）；
- 需要预防用药≤24小时的情况。

此时应停止研究药物输液。一旦研究输注反应已经缓解或者严重程度降至1级及以下，就可以先前速度的约50%重新开始输液，并且密切监测任何恶化情况。

- 3级或4级：中度或危及生命
- 3级：

1. 治疗后延迟起效（例如：对症用药和/或暂时中断输液后没有快速起效）；
2. 初期改善后症状复发；
3. 需要入院治疗临床后遗症。

- 4级：有危及生命的后果，需要紧急介入治疗；

此时，受试者必须立刻停止研究药物输液，并永久停止研究药物治疗。

若试验药物输液速度因输液相关反应而降低至先前50%，则后期治疗过程中，输液速度也必须一直按照先前输液速度50%进行。

# 参考文献

1. Bertolini F, Spallanzani A, Fontana A et al. Brain metastases: an overview. CNS Oncol 2015; 4: 37-46.

2. Tsukada Y, Fouad A, Pickren JW, Lane WW. Central nervous system metastasis from breast carcinoma. Autopsy study. Cancer 1983; 52: 2349-2354.

3. Patanaphan V, Salazar OM, Risco R. Breast cancer: metastatic patterns and their prognosis. South Med J 1988; 81: 1109-1112.

4. Santa-Maria CA, Nye L, Mutonga MB et al. Management of Metastatic HER2-Positive Breast Cancer: Where Are We and Where Do We Go From Here? Oncology (Williston Park) 2016; 30: 148-155.

5. Bartsch R, Berghoff AS, Preusser M. Breast cancer brain metastases responding to primary systemic therapy with T-DM1. J Neurooncol 2014; 116: 205-206.

6. Jacot W, Pons E, Frenel JS et al. Efficacy and safety of trastuzumab emtansine (T-DM1) in patients with HER2-positive breast cancer with brain metastases. Breast Cancer Res Treat 2016; 157: 307-318.

7. Fabi A, Alesini D, Valle E et al. T-DM1 and brain metastases: Clinical outcome in HER2-positive metastatic breast cancer. Breast 2018; 41: 137-143.

8. Montemurro F, Delaloge S, Barrios CH et al. Trastuzumab emtansine (T-DM1) in patients with HER2-positive metastatic breast cancer and brain metastases: exploratory final analysis of cohort 1 from KAMILLA, a single-arm phase IIIb clinical trial(☆). Ann Oncol 2020; 31: 1350-1358.

9. Bachelot T, Romieu G, Campone M et al. Lapatinib plus capecitabine in patients with previously untreated brain metastases from HER2-positive metastatic breast cancer (LANDSCAPE): a single-group phase 2 study. Lancet Oncol 2013; 14: 64-71.

10. Petrelli F, Ghidini M, Lonati V et al. The efficacy of lapatinib and capecitabine in HER-2 positive breast cancer with brain metastases: A systematic review and pooled analysis. Eur J Cancer 2017; 84: 141-148.

11. Lin NU, Lee EQ, Aoyama H et al. Response assessment criteria for brain metastases: proposal from the RANO group. Lancet Oncol 2015; 16: e270-278.
